# Mutual learning for joint disease detection and severity prediction reveals multimodal pathogenesis for neurodegenerative disorders

**DOI:** 10.1093/bioinformatics/btaf629

**Published:** 2025-12-27

**Authors:** Jin Zhang, Yixin Ji, Jinhua Liu, Wenrui Cui, Xiaohui Yao, Hongdong Li, Daoqiang Zhang, Lei Du, Alzheimer’s Disease Neuroimaging Initiative, Michael Weiner, Paul Aisen, Ronald Petersen, Michael Weiner, Paul Aisen, Ronald Petersen, Clifford R Jack, William Jagust, Susan Landau, Monica Rivera-Mindt, Ozioma Okonkwo, Leslie M Shaw, Edward B Lee, Arthur W Toga, Laurel Beckett, Danielle Harvey, Robert C Green, Andrew J Saykin, Kwangsik Nho, Richard J Perrin, Duygu Tosun, Pallavi Sachdev, Robert C Green, Erin Drake, Tom Montine, Cat Conti, Michael W Weiner, Rachel Nosheny, Diana Truran, Juliet Fockler, Melanie J Miller, Catherine (Cat) Conti, Winnie Kwang, Chengshi Jin, Adam Diaz, Miriam Ashford, Derek Flenniken, Adrienne Kormos, Ronald Petersen, Paul Aisen, Michael Rafii, Rema Raman, Gustavo Jimenez, Michael Donohue, Jennifer Salazar, Andrea Fidell, Virginia Boatwright, Justin Robison, Caileigh Zimmerman, Yuliana Cabrera, Sarah Walter, Taylor Clanton, Elizabeth Shaffer, Caitlin Webb, Lindsey Hergesheimer, Stephanie Smith, Sheila Ogwang, Olusegun Adegoke, Payam Mahboubi, Jeremy Pizzola, Cecily Jenkins, Laurel Beckett, Danielle Harvey, Michael Donohue, Naomi Saito, Adam Diaz, Kedir Adem Hussen, Ozioma Okonkwo, Monica Rivera-Mindt, Hannatu Amaza, Mai Seng Thao, Shaniya Parkins Mt Sinai, Omobolanle Ayo, Matt Glittenberg, Isabella Hoang, Kaori Kubo Germano, Joe Strong, Trinity Weisensel, Fabiola Magana, Lisa Thomas, Vanessa Guzman, Adeyinka Ajayi, Joseph Di Benedetto, Sandra Talavera, Clifford R Jack, Jr ,, Joel Felmlee, Nick C Fox, Paul Thompson, Charles DeCarli, Arvin Forghanian-Arani, Bret Borowski, Calvin Reyes, Caitie Hedberg, Chad Ward, Christopher Schwarz, Denise Reyes, Jeff Gunter, John Moore-Weiss, Kejal Kantarci, Leonard Matoush, Matthew Senjem, Prashanthi Vemuri, Robert Reid, Ian Malone, Sophia I Thomopoulos, Talia M Nir, Neda Jahanshad, Alexander Knaack, Evan Fletcher, Danielle Harvey, Duygu Tosun-Turgut, Stephanie Rossi Chen, Mark Choe, Karen Crawford, Paul A Yushkevich, Sandhitsu Das, William Jagust, Susan Landau, Robert A Koeppe, Gil Rabinovici, Victor Villemagne, Brian LoPresti, Richard J Perrin, John Morris, Erin Franklin, Haley Bernhardt, Nigel J Cairns, Lisa Taylor-Reinwald, Leslie Shaw, Edward B Lee, Virginia M Y Lee, Magdalena Korecka, Magdalena Brylska, Yang Wan, J Q Trojanowki, Arthur W Toga, Karen Crawford, Scott Neu, Andrew J Saykin, Kwangsik Nho, Tatiana M Foroud, Taeho Jo, Shannon L Risacher, Hannah Craft, Liana G Apostolova, Kelly Nudelman, Kelley Faber, Zoë Potter, Kaci Lacy, Rima Kaddurah-Daouk, Li Shen, David Soleimani-Meigooni, Renaud La Joie, Konstantinos Chiotis, Maison Abu Raya, Agathe Vrillon, Charles Windon, Julien Lagarde, Zoe Lin, Aidyn Rose Hills, Jason Karlawish, Claire Erickson, Joshua Grill, Emily Largent, Kristin Harkins, Michael W Weiner, Leon Thal, Zaven Kachaturian, Richard Frank, Peter J Snyder, Neil Buckholtz, John K Hsiao, Laurie Ryan, Susan Molchan, Zaven Khachaturian, Maria Carrillo, William Potter, Lisa Barnes, Marie Bernard, Hector González, Carole Ho Denali, John K Hsiao, Jonathan Jackson, Eliezer Masliah, Donna Masterman, Ozioma Okonkwo, Richard Perrin, Laurie Ryan, Nina Silverberg, Lisa Silbert, Jeffrey Kaye, Sylvia White (Salazar), Aimee Pierce, Amy Thomas, Tera Clay, Daniel Schwartz, Gillian Devereux, Janet "Janae" Taylor, Jennifer Ryan, Mike Nguyen, Madison DeCapo, Yanan Shang, Lon Schneider, Cynthia Munoz, Diana Ferman, Carlota Conant, Katherin Martin, Kristin Oleary, Sonia Pawluczyk, Elizabeth Trejo, Karen Dagerman, Liberty Teodoro, Mauricio Becerra, Madiha Fairooz, Sonia Garrison, Julia Boudreau, Yair Avila, James Brewer, Aaron Jacobson, Antonio Gama, Chi Kim, Emily Little, Jennifer Frascino, Nichol Ferng, Socorro Trujillo, Judith Heidebrink, Robert Koeppe, Steven MacDonald, Dariya Malyarenko, Jaimie Ziolkowski, James O’Connor, Nicole Robert, Suzan Lowe, Virginia Rogers, Ronald Petersen, Barbara Hackenmiller, Bradley Boeve, Colleen Albers, Connie Kreuger, David Jones, David Knopman, Hugo Botha, Jessica Magnuson, Jonathan Graff-Radford, Kerry Crawley, Michael Schumacher, Sanna McKinzie, Steven Smith, Tascha Helland, Val Lowe, Vijay Ramanan, Valory Pavlik, Jacob Faircloth, Jeffrey Bishop, Jessica Nath, Maria Chaudhary, Maria Kataki, Melissa Yu, Nathiel Pacini, Randall Barker, Regan Brooks, Ruchi Aggarwal, Lawrence Honig, Yaakov Stern, Akiva Mintz, Jonathan Cordona, Michelle Hernandez, Justin Long, Abbey Arnold, Alex Groves, Anna Middleton, Blake Vogler, Cierra McCurry, Connie Mayo, Cyrus Raji, Fatima S Amtashar, Heather Klemp, Heather Nicole, James Ruszkiewicz, Jasmina Kusuran, Jasmine Stewart, Jennifer Horenkamp, Julia Greeson, Kara Wever, Katie Vo, Kelly Larkin, Lesley Rao, Lisa Schoolcraft, Lora Gallagher, Madeline Paczynski, Maureen McMillan, Michael Holt, Nicole Gagliano, Rachel Henson, Renee LaBarge, Robert Swarm, Sarah Munie, Serena Cepeda, Stacey Winterton, Stephen Hegedus, TaNisha Wilson, Tanya Harte, Zach Bonacorsi, David Geldmacher, Amber Watkins, Brandi Barger, Bryan Smelser, Charna Bates, Cynthia Stover, Emily McKinley, Gregory Ikner, Haley Hendrix, Harold Matthew Cooper, Jennifer Mahaffey, Lindsey Booth Robbins, Loren Brown Ashley, Marissa Natelson-Love, Veronika Solomon, Hillel Grossman, Alexandra Groome, Allison Ardolino, Anthony Kaplan, Faye Sheppard, Genesis Burgos-Rivera, Gina Garcia-Camilo, Joanne Lim, Judith Neugroschl, Kimberly Jackson, Kirsten Evans, Laili Soleimani, Mary Sano, Nasrin Ghesani, Sarah Binder, Xiomara Mendoza Apuango, Ajay Sood, Amelia Troutman, Kimberly Blanchard, Arlene Richards,, Grace Nelson, Kirsten Hendrickson, Erin Yurko, Jamie Plenge, Victoria Rufo, Raj Shah, Ranjan Duara, Brendan Lynch, Cesar Chirinos, Christine Dittrich, Debbie Campbell, Diego Mejia, Gilberto Perez, Helena Colvee, Joanna Gonzalez, Josalen Gondrez, Joshua Knaack, Mara Acevedo, Maria Cereijo, Maria Greig-Custo, Michelle Villar, Morris Wishnia, Sheryl Detling, Warren Barker, Marilyn Albert, Abhay Moghekar, Barbara Rodzon, Corey Demsky, Gregory Pontone, Jim Pekar, Leonie Farrington, Martin Pomper, Nicole Johnson, Tolulope Alo, Martin Sadowski, Anaztasia Ulysse, Arjun Masurkar, Brittany Marti, David Mossa, Emilie Geesey, Emily Petrocca, Evan Schulze, Jennifer Wong, Joseph Boonsiri, Sunnie Kenowsky, Tatianne Martinez, Veronica Briglall, P Murali Doraiswamy, Adaora Nwosu, Alisa Adhikari, Cammie Hellegers, Jeffrey Petrella, Olga James, Terence Wong, Thomas Hawk, Sanjeev Vaishnavi, Hannah McCoubrey, Ilya Nasrallah, Rachel Rovere, Jeffrey Maneval, Elizabeth Robinson, Francisco Rivera, Jade Uffelman, Martha Combs, Patricia O’Donnell, Sara Manning, Richard King, Alayne Nieto, Amanda Glueck, Anjana Mandal, Audrie Swain, Bethanie Gamble, Beverly Meacham, Denece Forenback, Dorothy Ross, Elizabeth Cheatham, Ellen Hartman, Gary Cornell, Jordan Harp, Laura Ashe, Laura Goins, Linda Watts, Morgan Yazell, Prabin Mandal, Regan Buckler, Sylvia Vincent, Triana Rudd, Oscar Lopez, Ann Arlene Malia, Caitlin Chiado, Cary Zik, James Ruszkiewicz, Kathleen Savage, Linda Fenice, MaryAnn Oakley, Paige C Tacey, Sarah Berman, Sarah Bowser, Stephen Hegedus, Xanthia Saganis, Anton Porsteinsson, Abigail Mathewson, Asa Widman, Bridget Holvey, Emily Clark, Esmeralda Morales, Iris Young, James Ruszkiewicz, Kevin Hopkins, Kimberly Martin, Nancy Kowalski, Rebecca Hunt, Roberta Calzavara, Russell Kurvach, Stephen D’Ambrosio, Gaby Thai, Beatriz Vides, Brigit Lieb, Catherine McAdams-Ortiz, Cyndy Toso, Ivan Mares, Kathryn Moorlach, Luter Liu, Maria Corona, Mary Nguyen, Melanie Tallakson, Michelle McDonnell, Milagros Rangel, Neetha Basheer, Patricia Place, Romina Romero, Steven Tam, Trung Nguyen, Abey Thomas, Alexander (Alex) Frolov, Alka Khera, Amy Browning, Brendan Kelley, Courtney Dawson, Dana Mathews, Elaine Most, Elizeva (Ellie) Phillips, Lynn Nguyen, Maribel Nunez, Matalin Miller, Matthew R Jones, Natalie Martinez, Rebecca Logan, Roderick McColl, Sari Pham, Tiffani Fox, Tracey Moore, Allan Levey, Abby Brown, Andrea Kippels, Ashton Ellison, Casie Lyons, Chadwick Hales, Cindy Parry, Courtney Williams, Elizabeth McCorkle, Guy Harris, Heather Rose, Inara Jooma, Jahmila Al-Amin, James Lah, James Webster, Jessica Swiniarski, Latasha Chapman, Laura Donnelly, Lauren Mariotti, Mary Locke, Phyllis Vaughn, Rachael Penn, Sallie Carpentier, Samira Yeboah, Sarah Basadre, Sarah Malakauskas, Stefka Lyron, Tara Villinger, Terra Burney, Jeffrey Burns, Ala Abusalim, Alexandra Dahlgren, Alexandria Montero, Anne Arthur, Heather Dooly, Katelynn Kreszyn, Katherine Berner, Lindsey Gillen, Maria Scanlan, Mercedes Madison, Nicole Mathis, Phyllis Switzer, Ryan Townley, Samantha Fikru, Samantha Sullivan, Ella Wright, Maryam Beigi, Anthony Daley, Ashley Ko, Brittney Luong, Glen Nyborg, Jessica Morales, Kelly Durbin, Lauren Garcia, Leila Parand, Lorena Macias, Lorena Monserratt, Maya Farchi, Pauline Wu, Robert Hernandez, Thao Rodriguez, Neill Graff-Radford, A’llana Marolt, Anton Thomas, Deborah Aloszka, Ercilia Moncayo, Erin Westerhold, Gregory Day, Kandise Chrestensen, Mary Imhansiemhonehi, Sanna McKinzie, Sochenda Stephens, Sylvia Grant, Jared Brosch, Amy Perkins, Aubree Saunders, Debra Silberberg Kovac, Heather Polson, Isabell Mwaura, Kassandra Mejia, Katherine Britt, Kathy King, Kayla Nichols, Kayley Lawrence, Lisa Rankin, Martin Farlow, Patricia Wiesenauer, Robert Bryant, Scott Herring, Sheryl Lynch, Skylar Wilson, Traci Day, William Korst, Christopher van Dyck, Adam Mecca, Alyssa Miller, Amanda Brennan, Amber Khan, Audrey Ruan, Carol Gunnoud, Chelsea Mendonca, Danielle Raynes-Goldfinger, Elaheh Salardini, Elisa Hidalgo, Emma Cooper, Erawadi Singh, Erin Murphy, Jeanine May, Jesse Stanhope, Jessica Lam, Julia Waszak, Kimberly Nelsen, Kimberly Sacaza, Mayer Joshua Hasbani, Meghan Donahue, Ming-Kai Chen, Nicole Barcelos, Paul Eigenberger, Robin Bonomi, Ryan O’Dell, Sarah Jefferson, Siddharth Khasnavis, Stephen Smilowitz, Susan DeStefano, Susan Good, Terry Camarro, Vanessa Clayton, Yanis Cavrel, YuQuan "Oliver" Lu, Howard Chertkow, Howard Bergman, Chris Hosein, Sandra Black, Anish Kapadia, Aparna Bhan, Benjamin Lam, Christopher Scott, Gillian Gabriel, Jennifer Bray, Ljubica Zotovic, Maria Samira Gutierrez, Mario Masellis, Marjan Farshadi, Maurylette Gui, Meghan Mitchell, Rebecca Taylor, Ruby Endre, Zhala Taghi-Zada, Robin Hsiung, Carolyn English, Ellen Kim, Eugene Yau, Haley Tong, Laura Barlow, Lauren Jennings, Michele Assaly, Paula Nunes, Tahlee Marian, Andrew Kertesz, John Rogers, Dick Trost, Dylan Wint, Charles Bernick, Donna Munic, Ian Grant, Aaliyah Korkoyah, Ali Raja, Allison Lapins, Caila Ryan, Jelena Pejic, Kailey Basham, Leena Lukose, Loreece Haddad, Lucas Quinlan, Nathaniel Houghtaling, Carl Sadowsky, Walter Martinez, Teresa Villena, Brigid Reynolds, Angelica Forero, Carolyn Ward, Emma Brennan, Esteban Figueroa, Giuseppe Esposito, Jessica Mallory, Kathleen Johnson, Kathryn Turner, Katie Seidenberg, Kelly McCann, Margaret Bassett, Melanie Chadwick, Raymond Scott Turner, Robin Bean, Saurabh Sharma, Gad Marshall, Aferdita Haviari, Alison Pietras, Bradley Wallace, Catherine Munro, Gladiliz Rivera-Delpin, Hadley Hustead, Isabella Levesque, Jennifer Ramirez, Karen Nolan, Kirsten Glennon, Mariana Palou, Michael Erkkinen, Nicole DaSilva, Pamela Friedman, Regina M Silver, Ricardo Salazar, Roxxanne Polleys, Scott McGinnis, Seth Gale, Tia Hall, Tuan Luu, Steven Chao, Emmeline Lin, Jaila Coleman, Kevin Epperson, Minal Vasanawala, Alireza Atri, Amy Rangel, Brittani Evans, Candy Monarrez, Carol Cline, Carolyn Liebsack, Daniel Bandy, Danielle Goldfarb, Debbie Intorcia, Jennifer Olgin, Kelly Clark, Kelsey King, Kylee York, Marina Reade, Michael Callan, Michael Glass, Michaela Johnson-ACNP, Michele Gutierrez, Molly Goddard, Nadira Trncic, Parichita Choudhury, Priscilla Reyes, Serena Lowery, Shaundra Hall, Sonia Olgin, Stephanie de Santiago, Michael Alosco, Alyssa Ton, Amanda Jimenez, Andrew Ellison, Anh Tran, Brandon Anderson, Della Carter, Donna Veronelli, Steven Lenio, Eric Steinberg, Jesse Mez, Jason Weller, Jennifer Johns, Jesse Mez, Jessica Harkins, Alexa Puleio, Ina Hoti, Jane Mwicigi, Alexa Puleio, Michael Alosco, Olivia Schultz, Mona Lauture, Eric Steinberg, Ridiane Denis, Ronald Killiany, Sarab Singh, Steven Lenio, Wendy Qiu, Ycar Devis, Thomas Obisesan, Andrew Stone, Debra Ordor, Ifreke Udodong, Immaculata Okonkwo, Javed Khan, Jillian Turner, Kyliah Hughes, Oshoze Kadiri, Charles Duffy, Ariana Moss, Katherine Stapleton, Maria Toth (fmr Gross), Marianne Sanders, Martin Ayres, Melissa Hamski, Parianne Fatica, Paula Ogrocki, Sarah Ash, Stacy Pot, Doris Chen, Andres Soto, Costin Tanase, David Bissig, Hafsanoor Vanya, Heather Russell, Hitesh Patel, Hongzheng Zhang, Kelly Wallace, Kristi Ayers, Maria Gallegos, Martha Forloines, Meghan Sinn, Queennie Majorie S Kahulugan, Richard Isip, Sandra Calderon, Talia Hamm, Michael Borrie, T-Y Lee, Rob Bartha, Sterling Johnson, Sanjay Asthana, Cynthia M Carlsson, Allison Perrin, Pierre Tariot, Adam Fleisher, Stephanie Reeder, Horacio Capote, Allison Emborsky, Anna Mattle, Bela Ajtai, Benjamin Wagner, Bennett Myers, Daryn Slazyk, Delaney Fragale, Erin Fransen, Heather Macnamara, Jonathan Falletta, Joseph Hirtreiter, Laszlo Mechtler, Megan King, Michael Asbach, Michelle Rainka, Richard Zawislak, Scott Wisniewski, Stephanie O’Malley, Tatiana Jimenez-Knight, Todd Peehler, Traci Aladeen, Vernice Bates, Violet Wenner, Wisam Elmalik, Douglas W Scharre, Arun Ramamurthy, Soumya Bouchachi, Maria Kataki, Rawan Tarawneh, Brendan Kelley, Dzintra Celmins, Alicia Leader, Chris Figueroa, Heather Bauerle, Katlynn Patterson, Michael Reposa, Steven Presto, Tuba Ahmed, Wendy Stewart, Godfrey D Pearlson MD, Karen Blank, Karen Anderson, Robert B Santulli, Eben S Schwartz, Jeff Williamson, Alicia Jessup, Andrea Williams, Crystal Duncan, Abigail O’Connell, Karen Gagnon, Ezequiel Zamora, James Bateman, Freda Crawford, Deb Thompson, Eboni Walker, Jennifer Rowell, Mikell White, Phillip "Hunter" Ledford, Sarah Bohlman, Susan Henkle, Joseph Bottoms, Lena Moretz, Bevan Hoover, Michael Shannon, Samantha Rogers, Wendy Baker, William Harrison, Chuang-Kuo Wu, Alexis DeMarco, Ava Stipanovich, Daniel Arcuri, Jan Clark, Jennifer Davis, Kerstin Doyon, Marie Amoyaw, Mauro Veras Acosta, Ronald Bailey, Scott Warren, Terry Fogerty, Victoria Sanborn, Meghan Riddle, Stephen Salloway, Paul Malloy, Stephen Correia, Charles Windon, Morgan Blackburn, Howard J Rosen, Bruce L Miller, Amanda Smith, Ijeoma Mba, Jenny Echevarria, Juris Janavs, Emily Roglaski, Meagan Yong, Rebecca Devine, Hamid Okhravi, Edgardo Rivera, Teresa Kalowsky, Caroline Smith, Christina Rosario, Joseph Masdeu, Richard Le, Maushami Gurung, Marwan Sabbagh, Angelica Garcia, Micah Ellis Slaughter, Nadeen Elayan, Skieff Acothley, Nunzio Pomara, Raymundo Hernando, Vita Pomara, Chelsea Reichert, Olga Brawman-Mintzer, Allison Acree, Arthur Williams, Campbell Long, Rebecca Long, Paul Newhouse, Sydni Jenee Hill, Amy Boegel, Sudha Seshadri, Amy Saklad, Floyd Jones, William Hu, V Sotelo, Yaneicy Gonazalez Rojas, Jacobo Mintzer, Crystal Flynn Longmire, Kenneth Spicer

**Affiliations:** Department of Intelligent Science and Technology, Northwestern Polytechnical University, Xi'an, 710072, China; School of Artificial Intelligence, Nanjing University of Aeronautics and Astronautics, Nanjing, 210000, China; MedVisAl Lab, Lee Kong Chian School of Medicine, Nanyang Technological University, Singapore, 308232, Singapore; Department of Intelligent Science and Technology, Northwestern Polytechnical University, Xi'an, 710072, China; Qingdao Innovation and Development Center, Harbin Engineering University, Qingdao, 266000, China; College of Intelligent Systems Science and Engineering, Harbin Engineering University, Harbin, 150001, China; School of Computer Science and Engineering, Central South University, Changsha, 410083, China; Hunan Provincial Key Lab on Bioinformatics, Central South University, Changsha, 410083, China; School of Artificial Intelligence, Nanjing University of Aeronautics and Astronautics, Nanjing, 210000, China; Department of Intelligent Science and Technology, Northwestern Polytechnical University, Xi'an, 710072, China

## Abstract

**Motivation:**

Neurodegenerative disorders influence millions of people worldwide, and uncovering the pathogenesis is of urgent need. Many efforts have been made to detect or predict neurodegenerative disorders, while exploring the pathogenesis has been ignored from a systemic perspective.

**Results:**

To handle this issue, we propose a novel and powerful method, referred to as Pathogenesis-aware Mutual-Assistance Classification and Regression Optimization (Pa-MACRO). First, Pa-MACRO incorporates a mutual-assistance bidirectional mapping technique with a joint-embedding fine-grained interpretability module. This can extract the intrinsic factors and their interactions of multimodal pathogenesis. Second, our method can simultaneously classify an at-risk individual and predict the severity triggered by neurodegenerative disorders. Furthermore, to address the small sample size issue and the high-dimensional issue, we meticulously incorporate a semi-supervised cooperative learning method to integrate unlabeled data and extend it to a chromosome-wide setting in the spirit of divide-and-conquer. The Alzheimer’s Disease Neuroimaging Initiative (ADNI) database was used to evaluate Pa-MACRO. Without bells and whistles, Pa-MACRO establishes new state-of-the-art results in various settings while maintaining superior interpretability, verifying its power and versatility in revealing the pathogenesis of neurodegenerative disorders.

**Availability and implementation:**

The software is publicly available at https://github.com/ZJ-Techie/Pa-MACRO.

## 1 Introduction

### 1.1 Background and motivation

Neurodegenerative disorders are quite complex and are characterized by multiple pathological alterations in the brain. They may be influenced by genetic variations, proteomic expressions, and environmental factors, ultimately manifesting as brain imaging changes. While extensive efforts have been made to make diagnosis and prediction using brain imaging data (Bakkouri and Afdel 2020, 2023, Lu *et al.* 2021, Smith *et al.* 2021, Yu *et al.* 2021, Bakkouri *et al.* 2022, Wen *et al.* 2024), this single-aspect approach overlooks the contributions of genetics, environmental factors, proteomics factors, and their interactions in shaping intermediate imaging phenotypes. Consequently, it lacks a systems-level perspective for developing effective strategies to slow down or even halt disease advancement.

### 1.2 Advances and limitations in imaging genetics

Brain imaging genetics offers a powerful avenue for uncovering the genetic basis of brain structure and function by jointly analyzing genetic variants, such as single nucleotide polymorphisms (SNPs) and neuroimaging-derived quantitative traits (QTs). This integrative approach has deepened our understanding of both typical and atypical brain processes. Over the past decade, a range of univariate, multivariate, and bi-multivariate methods have been developed to probe the genetic architecture of imaging endophenotypes (Shen and Thompson 2020, Huang *et al.* 2021, Yang *et al.* 2024, Zhang *et al.* 2024a, Wang *et al.* 2025). However, emerging evidence indicates that these endophenotypes are not solely shaped by additive genetic effects but are also influenced by gene–environment (G × E) interactions and gene–protein correlations. These interactions can modulate intermediate phenotypes and ultimately impact disease risk. Capturing both interaction and main effects is therefore critical for improving disease prediction and elucidating the heritability of brain disorders (Huang *et al.* 2021, Yu *et al.* 2024, Peng *et al.* 2025, Zhang *et al.* 2025a).

### 1.3 Challenges in joint disease detection and biomarker discovery

Nowadays, accurate disease detection, severity assessment, and pathogenesis discovery are fundamental yet challenging tasks for neurodegenerative disorders (Bakkouri and Afdel 2019, Shen and Thompson 2020, Bakkouri and Bakkouri 2024, 2025). However, (i) most existing studies address diagnosis and prediction separately, ignoring their strong interdependence. In reality, diagnostic categories reflect disease severity, which is closely linked to clinical outcomes. Jointly learning both tasks could leverage the intrinsic associations between clinical variables and categories, thereby enhancing detection/assessment performance (Andrews *et al.* 2019). (ii) Traditional approaches treat disease prediction and biomarker discovery as independent tasks. While disease prediction models aim to provide accurate diagnoses or disease severity prediction, biomarker discovery focuses on identifying accurate factors associated with disease (DeGroat *et al.* 2023, Guo *et al.* 2024, Zhang *et al.* 2024b, Zhong *et al.* 2025a). Treating them in isolation disregards their mutual reinforcement: interpretable biomarkers can enhance model transparency, and predictive models can guide meaningful feature selection. Although recent studies have advanced learning strategies for accurate prediction, they often lack intrinsic interpretability (Shen and Thompson 2020, Liu *et al.* 2024, Zhang *et al.* 2025b, Zhong *et al.* 2025b).

### 1.4 Motivation for a unified and interpretable framework

We thus advocate for a unified framework that jointly performs disease prediction and mechanistic inference to achieve both diagnostic reliability and biological fine-grained interpretability (fine-grained interpretability means the model’s ability to quantitatively and qualitatively attribute predictive contributions to individual biomarkers). A further challenge in real-world applications is the scarcity of large-scale multi-omics datasets due to privacy constraints and the extended time required for data collection. Incorporating unlabeled data from extensive brain disorder datasets into a joint-embedding architecture presents a promising strategy to establish a valuable link between the wealth of underexploited data and researchers facing limited resources, thus promoting generalizability and reliability in disease prediction and biomarker identification.

### 1.5 Methodological and conceptual innovation

Unlike previous multimodal approaches that primarily capture statistical correlations among imaging, genomic, or proteomic modalities, the proposed Pa-MACRO framework establishes a mechanism-guided, multi-task co-learning paradigm that jointly optimizes disease diagnosis (classification), severity prediction (regression), and mechanistic biomarker discovery within a single end-to-end architecture. Through the *Mutual-Assistance (MA) Triad*—a three-stage cyclic learning process integrating multimodal representation learning, predictive modeling, and interpretable feature selection—Pa-MACRO enables mutual reinforcement between diagnostic and prognostic tasks while embedding interpretability directly into model training rather than relying on *post hoc* explanation. Furthermore, the integration of semi-supervised learning, divide-and-conquer chromosome-wide optimization, and fine-grained interpretability modules enhances scalability and biological reliability.

Our main scientific contributions are as follows:

- Drawing on the concept of Mutual Assistance (MA), we are the first to propose a robust yet practical joint classification and regression architecture with interpretable multimodal pathogenesis embeddings, termed Pa-MACRO. This unified framework effectively enhances disease diagnosis and quantitative prediction while facilitating biologically meaningful biomarker identification.
- We develop a novel mutual-assistance joint optimization paradigm that integrates genomics, environmental, proteomic, and radiomic information. By bridging micro-level molecular mechanisms and macro-level imaging phenotypes, the proposed model enables biologically informed downstream diagnostic classification and prediction.
- We further propose an iterative learning mechanism that allows disease prediction and biomarker identification to mutually reinforce each other, forming a synergistic loop that continuously improves both predictive accuracy and interpretability.
- To address the challenges of small sample sizes and high-dimensional multimodal data, we design a semi-supervised cooperative learning strategy that effectively leverages unlabeled data. Through a divide-and-conquer extension to chromosome-wide settings, our method achieves scalable and robust imaging-genetic modeling.
- Extensive experiments on different multi-omic medical datasets demonstrate the superiority, robustness, and interpretability of the proposed approach in neurodegenerative disease diagnosis and quantitative trait prediction, while enabling reliable biomarker discovery with clear biological relevance.

## 2 Materials and methods

### 2.1 The Pa-MACRO

**Figure 1.**
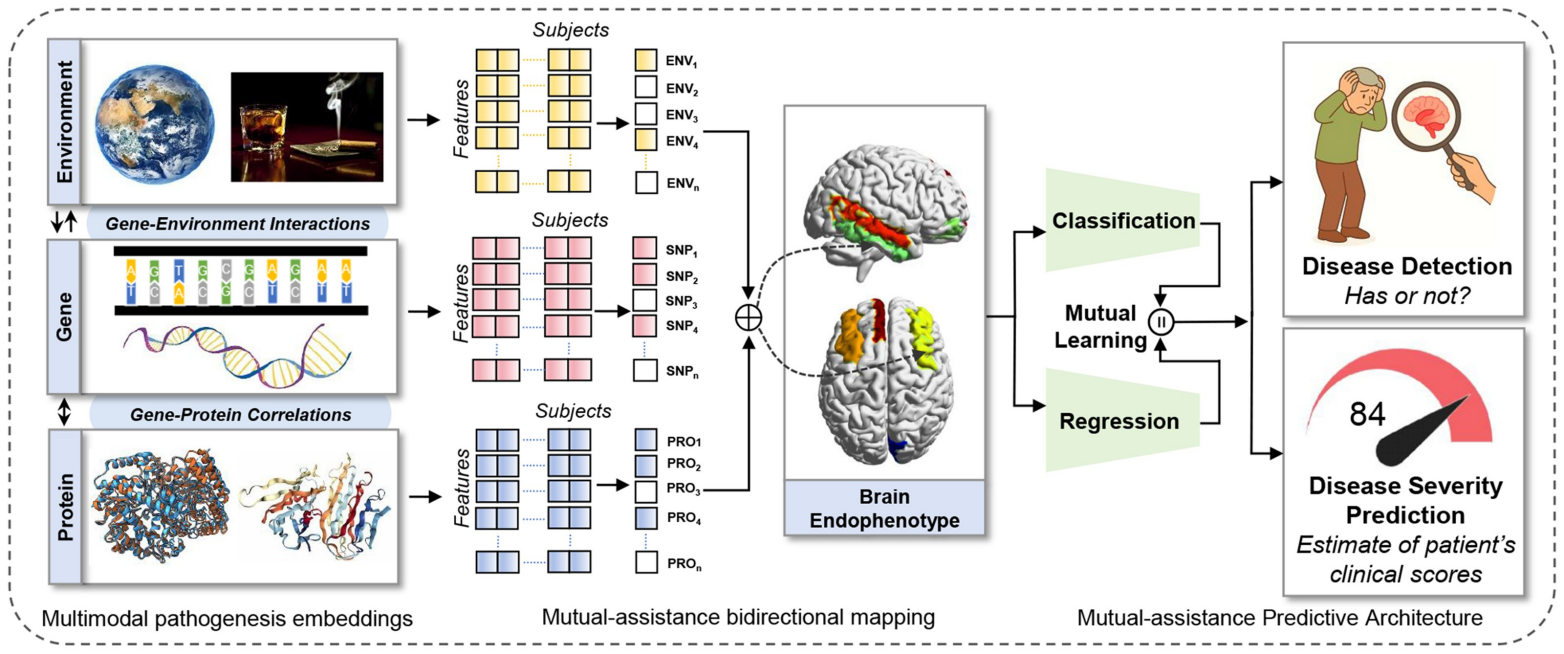
A schematic illustration of a mutual learning-assisted predictive architecture with interpretable multimodal pathogenesis embeddings. First, Pa-MACRO incorporates a mutual-assistance bidirectional mapping structure with joint-embedding fine-grained interpretability, which helps collaboratively extract the intrinsic unity and interactions of multimodal pathogenesis that contribute to imaging phenotypes. Second, we meticulously design a mutual-assistance joint classification/regression module to ensure joint detection/assessment, which promotes disease detection and severity prediction.

As mentioned above, we build on the concept of mutual-assistance learning to integrate multimodal representation, diagnosis/prediction, and biomarker identification into a unified framework, enabling both tasks to benefit from their intrinsic interdependence. To this end, we propose Pa-MACRO, a model specifically designed to facilitate the clinical translation of high-dimensional omics data by bridging the gap between predictive modeling and the sparsity and interpretability demands of clinical biomarker discovery. An overview of the Pa-MACRO architecture is presented in Fig. 1, which comprises three novel components: mutual-assistance joint bidirectional mapping (MAJBM), mutual-assistance joint classification/regression (MAJCR), and mutual-assistance prediction and discovery strategy.

#### 2.1.1 MA-1: unified mutual-assistance framework of representation learning and prediction modeling

First, the spirit of MA is manifested in trustworthy multimodal collaborative integration. Pa-MACRO incorporates a MA bidirectional mapping structure with the joint-embedding interpretability, which helps extract fine-grained factors and cross-modality alignment of interpretable genetic variations factors, environmental factors, genotype–environment interaction, proteomic factors, and disease-related imaging endophenotypes simultaneously, thus contributing to downstream diagnostic classification performance. Therefore, $L_{\text{MABM}}$ takes the form:


(1) $$\begin{array}{c} L_{\text{MABM}}=\sum_{i=1}^{n}{\left\|x_{i}^{T}u_{1}+x_{i}^{T}QE_{i}+c_{i}^{T}v_{1}-y_{i}^{T}w_{1}\right\|}_{2}^{2}\\ +\lambda_{1}\sum_{i=1}^{n}{\left\|x_{i}^{T}u_{2}-c_{i}^{T}v_{2}\right\|}_{2}^{2}+\lambda_{2}\sum_{i=1}^{n}{\left\|x^{\prime }{}_{i}^{T}u_{3}-c^{\prime }{}_{i}^{T}v_{3}\right\|}_{2}^{2} \end{array}$$

where $U=[u_{1},u_{2},u_{3}]$, $V=[v_{1},v_{2},v_{3}]$, and $W=[w_{1},w_{2},w_{3}]$ carry the main effects of SNPs, proteomic, and imaging markers, respectively, $Q\in R^{p_{1}\times p_{4}}$ carries the interaction effects between SNPs and environment markers, with promising evidence for pathogenesis discovery.

Of note, the SNPs are denoted as $X\in R^{n\times p_{1}}$, imaging phenotypes, proteins, and environmental exposures are denoted as $Y\in R^{n\times p_{2}}$, $C\in R^{n\times p_{3}}$, and $E\in R^{n\times p_{4}}$, z is clinical scores, and d is diagnostic status. *n* is the number of subjects. $p_{1}$, $p_{2}$, $p_{3}$, and $p_{4}$ represent the number of SNPs, imaging, proteomics biomarkers, and environmental exposures, respectively. Specifically, each modality (genetic, proteomic, environmental, and imaging) contributes to a common latent space via bidirectional mappings, enabling cross-modal correlation maximization and complementary information exchange. In addition, the unlabeled SNPs are denoted as $X^{\prime }\in R^{n_{\mathrm{unlabeled}}\times p_{1}}$, unlabeled proteins are denoted as $C^{\prime }\in R^{n_{\mathrm{unlabeled}}\times p_{3}}$. Of note, in the last part of $L_{\text{MABM}}$, semi-supervised cooperative learning (SCL) integrates unlabeled multi-omics to extract cross-modal co-expression patterns for improving detection/assessment, ensuring both stability and reliability, which is promising to establish a valuable link between the wealth of underexploited data and researchers facing limited resources.

#### 2.1.2 MA-2: mutual-assistance for disease detection and severity prediction

Second, the spirit of MA is also reflected in the joint optimization paradigm for disease diagnosis and prediction. Specifically, logistic regression is employed for disease classification, while multivariate least squares regression is used to predict clinical scores:


(2) $$\begin{array}{c} L_{\text{MAJCR}}\left(y_{i}^{T}w_{2},y_{i}^{T}w_{3},d,z\right)={\left\|Y^{T}w_{2}-z\right\|}_{F}^{2}\\ +\sum_{i=1}^{n}\sum_{k=1}^{F}\left(d_{ik}\;\text{log}\;\sum_{l=1}^{F}e^{w_{3}{}_{l}^{T}y_{i}}-d_{ik}w_{3}{}_{k}^{T}y_{i}\right)+\Omega \left(W\right) \end{array}$$

where *F* is the number of classes. $d_{ik}$ denotes the predicted disease status for subject *i* and class *k*. Here, we introduce a joint structured alignment regularizations between classification and regression features. This can enhance both features to work more collaboratively during the process of optimization, i.e. $\Omega (W)=\lambda_{w_{1}}||W|{|}_{2,1}+\lambda_{w_{2}}||W|{|}_{1,1}$, which ensures that the identified biomarkers are relevant to the disease of interest, finally mutually enhancing disease detection and assessment with greater flexibility and performance. Furthermore, to ensure reliable biomarker identification and facilitate interpretation, we introduce sparse variable regularizers (SVR) to select important features and remove unstable features, which could enhance interpretability while preserving predictability. We use $\Omega (Q)=||Q|{|}_{1,1}=\sum_{i}\sum_{j}\left|Q_{i,j}\right|$ to identify interpretable GE interactions. Ω(U) and Ω(V) control the sparsity of the main effects of genetics and proteomic markers. Following analogous joint optimization paradigm, we employ ${\text{FGL}}_{2,1}$-norm $\left({\left\|U\right\|}_{{\text{FGL}}_{2,1}}=\sum_{i=1}^{p-1}\sqrt{{\left\|u^{i}\right\|}_{2}^{2}+{\left\|u^{i+1}\right\|}_{2}^{2}}.\right)$, $\ell_{2,1}$-norm, and $\ell_{1}$-norm to identify significant SNPs at individual and group levels to understand the genetic architectures of ADs. Thus, $\Omega (U)=\lambda_{u_{1}}||U|{|}_{{\text{FGL}}_{2,1}}+\lambda_{u_{2}}||U|{|}_{2,1}+\lambda_{u_{3}}||U|{|}_{1,1}$. The FGL_2,1_ regularization captures both group-level correlations and local smoothness across neighboring features, enabling recovery of LD blocks while maintaining sparsity for interpretability. In addition, $\ell_{2,1}$-norm and $\ell_{1}$-norm are introduced to identify meaningful proteomics, i.e. $\Omega (V)=\lambda_{v_{1}}||V|{|}_{2,1}+\lambda_{v_{2}}||V|{|}_{1,1}$, giving Pa-MACRO better interpretability.

#### 2.1.3 MA-3: mutual-assistance for biomarker discovery and disease prediction

Third, the principle of mutual assistance is further embodied in the joint optimization paradigm for biomarker discovery and disease prediction. Unlike conventional *post hoc* interpretability methods, Pa-MACRO is designed as a fully end-to-end framework, enabling the identification of fine-grained explanatory features through a fully learnable process. We define $C=[c_{1},\ldots ,c_{n}]$ and $C^{\prime }=[c^{\prime }{}_{1},\ldots ,c^{\prime }{}_{n}]$. Likewise, X is the same as well. Together, these three components are synergistically integrated into a cohesive mutual-assistance triad framework (MA-3), as described below:


(3) $$\begin{array}{c} \underset{U,Q,W,V}{\min}\sum_{i=1}^{n}{\left\|x_{i}^{T}u_{1}+x_{i}^{T}QE_{i}+c_{i}^{T}v_{1}-y_{i}^{T}w_{1}\right\|}_{2}^{2}\\ +L_{R}\left(y_{i}^{T}w_{2},z\right)+L_{C}\left(y_{i}^{T}w_{3},d\right)\\ +\lambda_{1}\sum_{i=1}^{n}{\left\|x_{i}^{T}u_{2}-c_{i}^{T}v_{2}\right\|}_{2}^{2}+\lambda_{2}\sum_{i=1}^{n}{\left\|x^{\prime }{}_{i}^{T}u_{3}-c^{\prime }{}_{i}^{T}v_{3}\right\|}_{2}^{2}\\ \;s.t.\;||Xu_{k}|{|}_{2}^{2}=1,||Cv_{k}|{|}_{2}^{2}=1,||Yw_{k}|{|}_{2}^{2}=1,\\ \Omega (u_{k})\leq c_{u},\Omega (v_{k})\leq c_{v},\Omega (w_{k})\leq c_{w},\Omega (Q)\leq c_{Q} \end{array}$$

### 2.2 Extension to high-dimensional analysis

Further, the direct application of Pa-MACRO to high-dimensional analysis presents challenges due to the computational intensity of genotype matrices. To manage the high-dimensional SNPs and GE interactions, we partition them into *L* non-overlapping subsets, denoted as $U={⊕}_{l=1}^{L}U^{l}$ and $Q={⊕}_{l=1}^{L}Q^{l}$, respectively. We then adopt an efficient strategy that circumvents the direct computation of main and interaction terms by calculating these effects within each subset and subsequently combining the results across all genotypes. Following the divide-and-conquer principle, we reformulate Pa-MACRO as follows:


(4) $$\begin{array}{c} \underset{U,Q,V,W}{\min}\sum_{i=1}^{n}||x_{i}^{T}\left(u_{1_{1}}⊕\cdots ⊕u_{1_{L}}\right)+x_{i}^{T}\left(Q_{1}⊕\cdots ⊕Q_{L}\right)E_{i}\\ +c_{i}^{T}v_{1}-y_{i}^{T}w_{1}|{|}_{2}^{2}+L_{R}\left(y_{i}^{T}w_{2},z\right)\\ +L_{C}\left(y_{i}^{T}w_{3},d\right)+\lambda_{1}\sum_{i=1}^{n}{\left\|x_{i}^{T}\left(u_{2_{1}}⊕\ldots ⊕u_{2_{L}}\right)-c_{i}^{T}v_{2}\right\|}_{2}^{2}\\ +\lambda_{2}\sum_{i=1}^{n}{\left\|x^{\prime }{}_{i}^{T}\left(u_{3_{1}}⊕\ldots ⊕u_{3_{L}}\right)-c^{\prime }{}_{i}^{T}v_{3}\right\|}_{2}^{2}\\ \;s.t.\;||Xu_{k}|{|}_{2}^{2}=1,||Cv_{k}|{|}_{2}^{2}=1,||Yw_{k}|{|}_{2}^{2}=1,\Omega (U)\leq c_{u},\\ \Omega (V)\leq c_{v},\Omega (W)\leq c_{w},\Omega (Q)\leq c_{Q} \end{array}$$

where $L_{R}\left(y_{i}^{T}w_{2},z\right)$ and $L_{C}\left(y_{i}^{T}w_{3},d\right)$ represents regression and classification, respectively. The operator ⊕ represents the merging of SNPs and interaction terms. This decoupling allows for parallel processing, as SNPs and interaction terms can be processed independently, reducing memory requirements. Fast Pa-MACRO only needs to store small SNP matrices during iteration. As shown in Equation (4), Pa-MACRO is multi-convex and can be optimized using an alternating convex search (ACS) strategy. We first fix V, Q, and W to solve for U using gradient descent and then iteratively update each variable, guaranteeing the convergence to a local optimum.

Algorithm 1. The Pa-MACRO algorithm

Require

The SNPs $X\in R^{n\times p_{1}}$, imaging phenotypes, proteins and environmental exposures $Y\in R^{n\times p_{2}}$, $C\in R^{n\times p_{3}}$, and $E\in R^{n\times p_{4}}$, z clinical scores, d diagnosis category, as well as the tradeoff parameters.

**Ensure:** 

Canonical weights U, Q , V, and W.

**1:** Initialize $U\in R^{p_{1}\times 3}$, $Q\in R^{p_{1}\times p_{4}}$, $V\in R^{p_{2}\times 3}$, $W\in R^{p_{3}\times 3}$.

**2: while** not convergence **do** 

**3:** Solve U with gradient descent by fixing Q, V, and W.

**4:** Solve Q with gradient descent by fixing U, V, and W.

**5:** Solve V with gradient descent by fixing Q, U, and W.

**6:** Solve W with gradient descent by fixing U, Q, and V.

**7: end while** 

**8:** Sorting U, Q, V, and W based on their absolute values and reporting the user or domain expert-defined top biomarkers.

## 3. Results

### 3.1 Experimental setup

#### 3.1.1 State-of-the-art methods

We systematically evaluated Pa-MACRO against a series of representative and widely recognized imaging genetics techniques, including SMCCA, AdaSMCCA, and RelPMDCCA (Witten and Tibshirani 2009, Hu *et al.* 2018, Rodosthenous *et al.* 2020). By comparison, although many recent deep learning architectures demonstrated strong predictive performance, they inherently lacked interpretability. In particular, they often failed to isolate key explanatory variables from the original input domain—especially those capturing G × E interaction effects. Their dependence on *post hoc* interpretation methods consequently limited their effectiveness in elucidating the biological mechanisms underlying complex disease processes.

#### 3.1.2 Evaluation criteria and parameter setting

The experimental evaluation was based on three key metrics: the quality of selected feature subsets, the accuracy of clinical score prediction, and the effectiveness of disease status classification. To ensure robust and unbiased model tuning, we employed nested five-fold cross-validation, systematically exploring a hyperparameter search space defined as $10^{i}$ (i=−4,−3,−2,…,0,…,2,3,4), selecting parameters that produced the highest mean testing performance.

### 3.2 Results on the Alzheimer’s disease dataset

#### 3.2.1 Dataset

The dataset comprised 244 subjects, including 42 healthy controls (HC), 137 individuals with mild cognitive impairment (MCI), and 65 patients diagnosed with AD, all obtained from the Alzheimer’s Disease Neuroimaging Initiative (ADNI) database (adni.loni.usc.edu). Multimodal data sources encompassed: (i) proteomic profiles from cerebrospinal fluid and blood plasma, yielding 146 protein markers post quality control via the rules-based medicine (RBM) platform; (ii) quantitative imaging traits (QTs) derived from voxel-based morphometry (VBM) of structural MRI; and (iii) cortical and subcortical morphometric features, including volumetric and thickness measures extracted using FreeSurfer. In addition, 16 environmental risk variables were incorporated, such as age, vision status, body mass index, alcohol consumption, drug sensitivity, blood pressure, smoking history, and prior stroke events. To enhance the generalizability of the model, unlabeled SNPs and protein data from large-scale multi-omics datasets were further integrated into our model to improve the robustness of disease prediction and biomarkers discovery. In total, 10 000 high-quality SNPs known risk loci such as *APOE*, *APOC1*, and *TOMM40* were retained for subsequent analyses. To assure a stable and reliable result, we normalized the data (*z*-scores) to remove the influence of data scales.

#### 3.2.2 Enhanced prediction of clinical scores and accurate disease status classification

**Figure 2.**
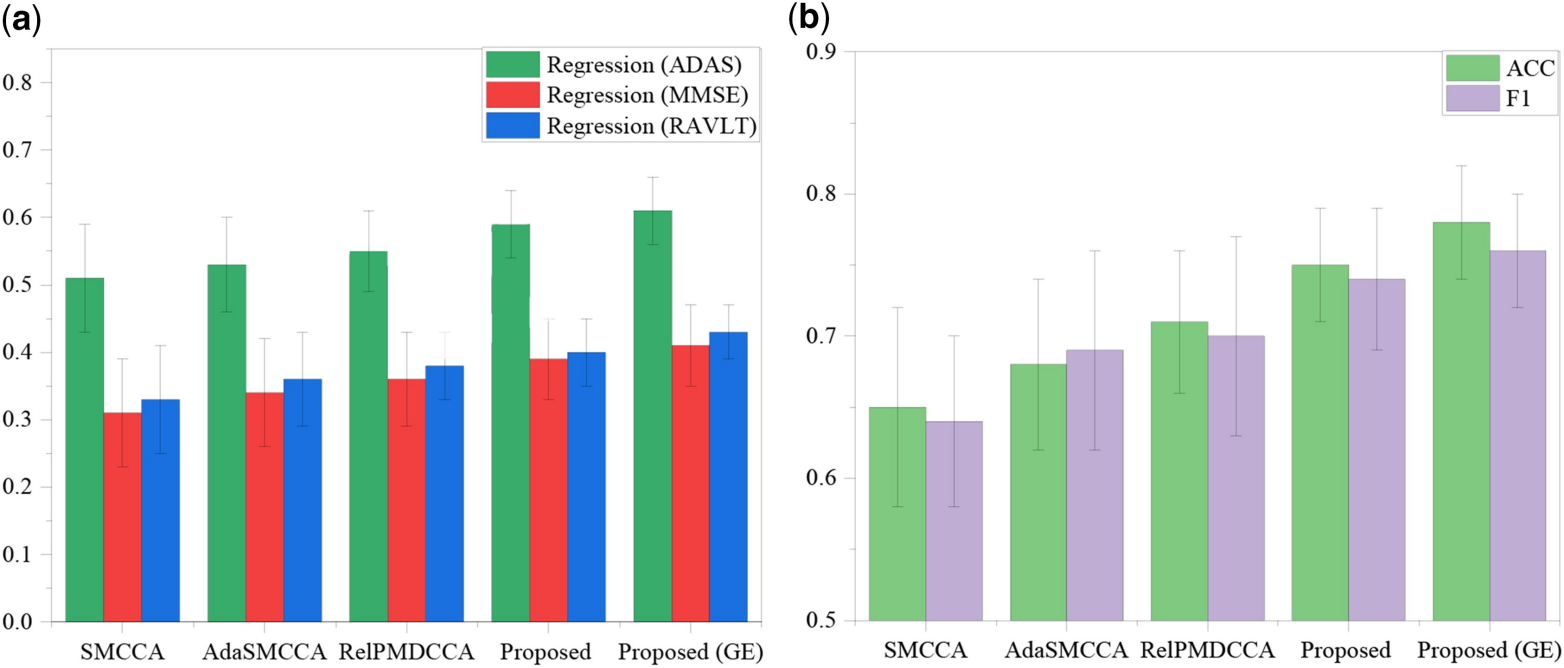
Comparison of evaluation outcomes for AD diagnosis (ACC, *F*1) and assessment (CCC) on VBM (a, b), where GE denotes inclusion of gene–environment interactions. Error bars indicate the standard deviation (SD) across five-fold cross-validation runs.

As shown in Fig. 2 a and b, we evaluated Pa-MACRO’s performance on cognitive score prediction and disease status classification. The metrics included correlation coefficients (CCCs) for regression tasks and accuracy (ACC) and *F*1 scores for classification tasks. Pa-MACRO consistently surpassed baseline models and joint classification–regression methods based on VBM, achieving superior classification accuracy and more robust cognitive score predictions across ADAS, MMSE, and RAVLT. These results highlighted the efficacy of the mutual-assistance learning paradigm in improving both diagnostic accuracy and severity. To validate the superiority of the proposed, we performed paired *t*-tests between Pa-MACRO and all benchmark approaches, and all comparisons reached statistical significance (*P* < .05).

#### 3.2.3 Multi-omics explanation (genetic, proteomic, and neuroimaging-derived phenotype)

**Figure 3.**
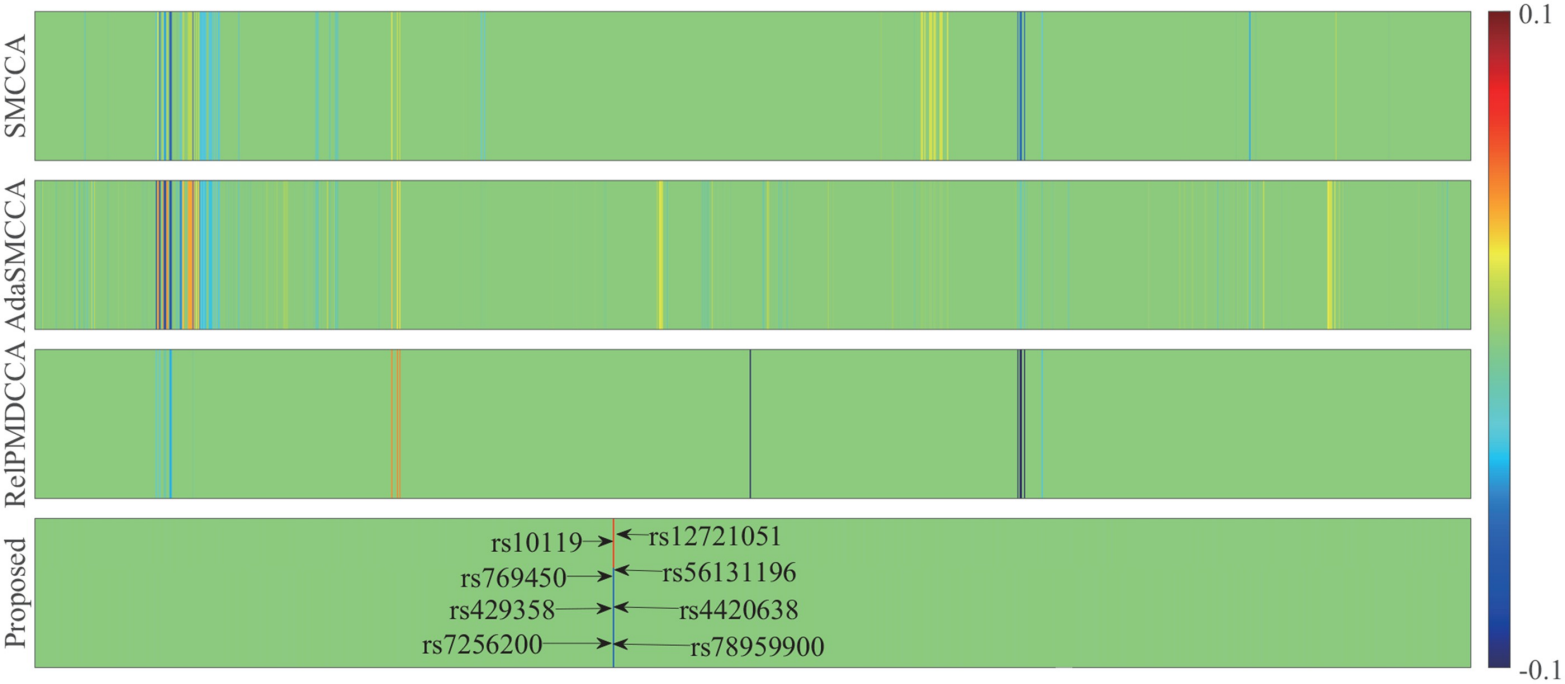
Average canonical weights of SNPs derived from five-fold cross-validation. Each row corresponds to a method: (i) SMCCA; (ii) AdaSMCCA; (iii) RelPMDCCA; and (iv) Proposed.

**Figure 4.**
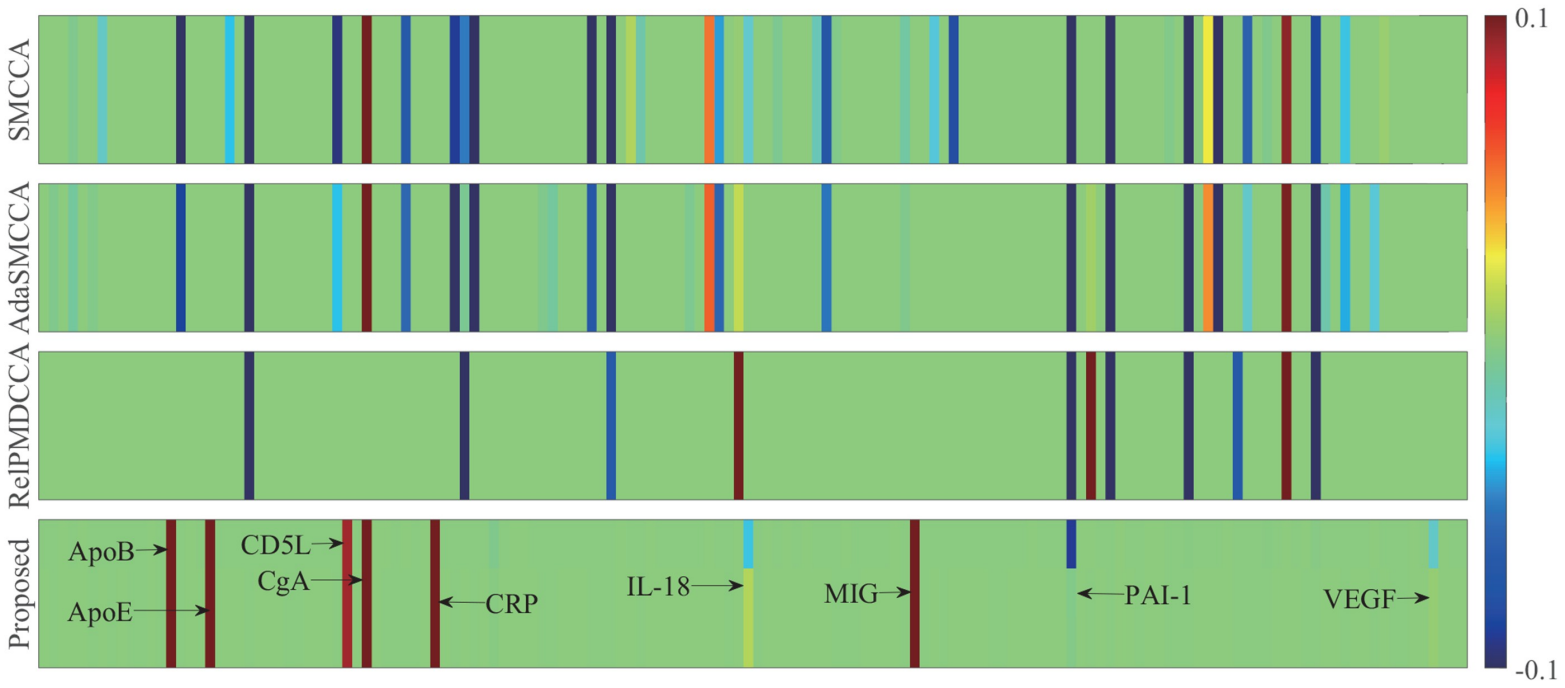
Average canonical coefficients of proteomic markers. Each row represents a method: (i) SMCCA; (ii) AdaSMCCA; (iii) RelPMDCCA; and (iv) Proposed.

**Figure 5.**
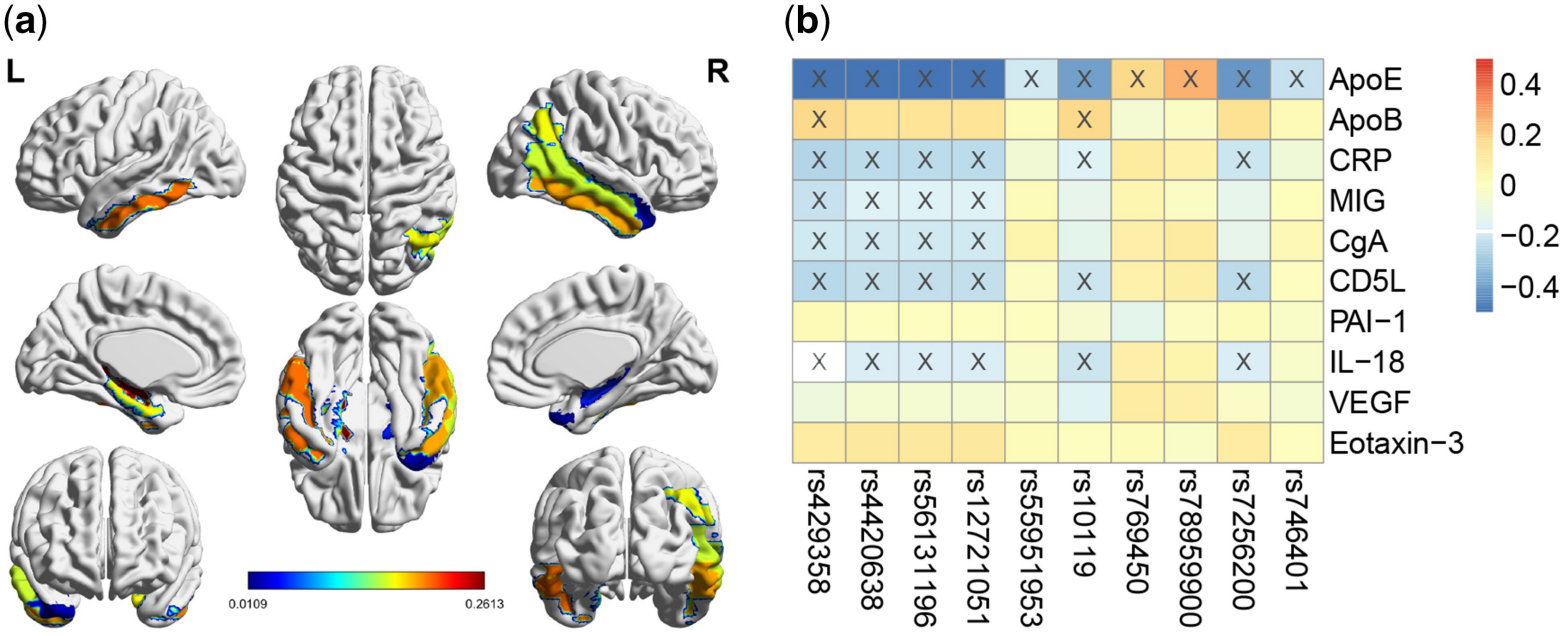
(a) Visualization of the detected brain imaging quantitative traits (QTs). (b) Heatmap depicting pairwise correlations between the top 10 SNPs and biomarkers, where the symbol “×” denotes statistically significant associations (*P* < .05).

Figure 3 illustrates the weight of SNPs, where Pa-MACRO successfully identified several AD-risk loci, including the well-established rs429358 (*APOE*), rs4420638 (*APOC*1), rs56131196 (*APOC*1), and rs12721051 (*APOC*1) (Serrano-Pozo *et al.* 2021). We then performed ANOVA to examine their main effects on the diagnostic phenotype. As expected, all *P*-values were statistically significant (*P* < .05). Due to the ${\text{FGL}}_{2,1}$-norm, meaningful SNP groups were identified, including rs4420638, rs56131196, and rs12721051 (*APOC*1), all with *P* = $1.88\times 10^{-9}$. These findings supported the oligogenic or polygenic nature of AD. Figures 4 and 5 demonstrates that Pa-MACRO successfully identified several AD-related proteomic markers (ApoE, ApoB, CRP, MIG, and CgA) (Serrano-Pozo *et al.* 2021), imaging phenotypes including the right and left hippocampus, parahippocampus, and temporal pole regions (Frisoni *et al.* 2008, Fan *et al.* 2019, Geigenmüller *et al.* 2024). In contrast, baselines generated numerous irrelevant signals that may introduce misleading information in downstream analyses.

Beyond the main effects, Pa-MACRO identified significant gene–environment interactions. The top five interactions were (rs12721046, smoking), (rs1160984, alcohol abuse), (rs79429216, tremor), (rs1160984, stroke), and (rs75654248, visual impairment). Notably, the rs12721046–smoking interaction was linked to AD, aligning with prior findings that rs12721046 was an AD-risk variant and that smoking contributed to disease detection (Serrano-Pozo *et al.* 2021), as the co-occurrence of these factors may enhance clinicians’ confidence in identifying at-risk individuals. We also conducted an ablation study by removing the GE interactions (Fig. 2). As expected, the best performance was achieved when both main and interaction effects were considered, highlighting the critical importance of GEs. The heatmap in Fig. 5b further illustrated SNP–protein interactions, where the (rs429358, ApoE) pair exhibited the strongest correlation, aligning with established evidence that rs429358 could play a crucial role in encoding the ApoE protein. These findings verified Pa-MACRO in identifying biologically meaningful AD-related biomarkers and facilitating targeted therapeutic interventions.

### 3.3 Follow-up analyses: phenome-wide association studies

**Figure 6.**
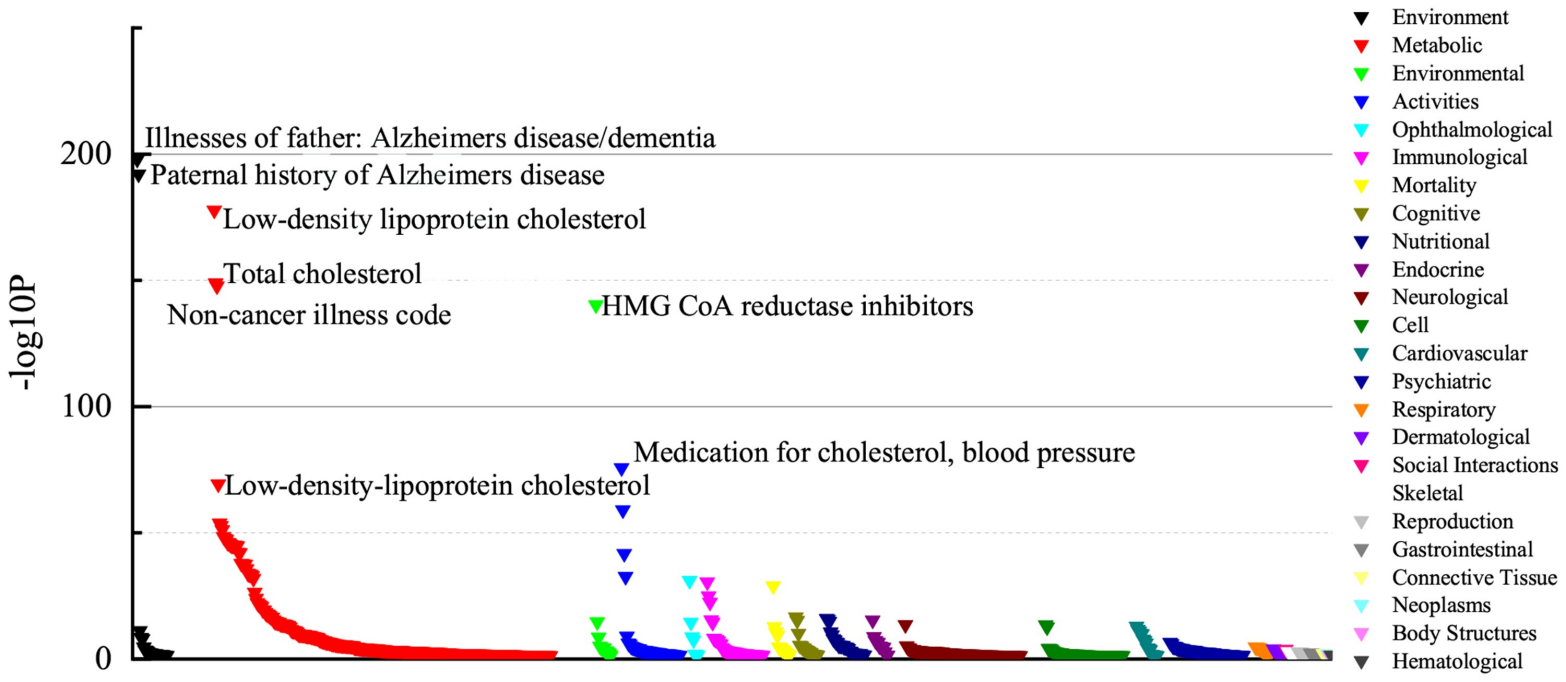
The PheWAS investigation produced findings for SNP rs4420638 (*APOC*1).

To validate the phenotypic relevance of SNPs identified by Pa-MACRO, we conducted a phenotype-wide association study (PheWAS) using publicly available data from the GWAS Atlas database (https://atlas.ctglab.nl), which aggregates results from 4756 GWAS. As shown in Fig. 6, rs4420638 demonstrated significant associations with several neurological traits, including Alzheimer’s disease and paternal family history of dementia. It was also linked to cardiometabolic conditions such as elevated blood pressure and diabetes. Similarly, *APOC*1 remained strongly associated with neurological phenotypes, reinforcing its established role in neurodegenerative susceptibility. These results validated the ability of Pa-MACRO to detect stable and biologically relevant genetic signals.

### 3.4 Correlation between imaging-based biomarkers and clinical cognitive

To facilitate clinical application of our findings, we examined the relationships between top neuroimaging-derived phenotypes and cognitive function in individuals at risk for AD. Specifically, we analyzed correlations between the identified imaging biomarkers and clinical cognitive measures, focusing on the Rey Auditory Verbal Learning Test (RAVLT), a widely used metric sensitive to AD-related cognitive impairment.

**Figure 7.**
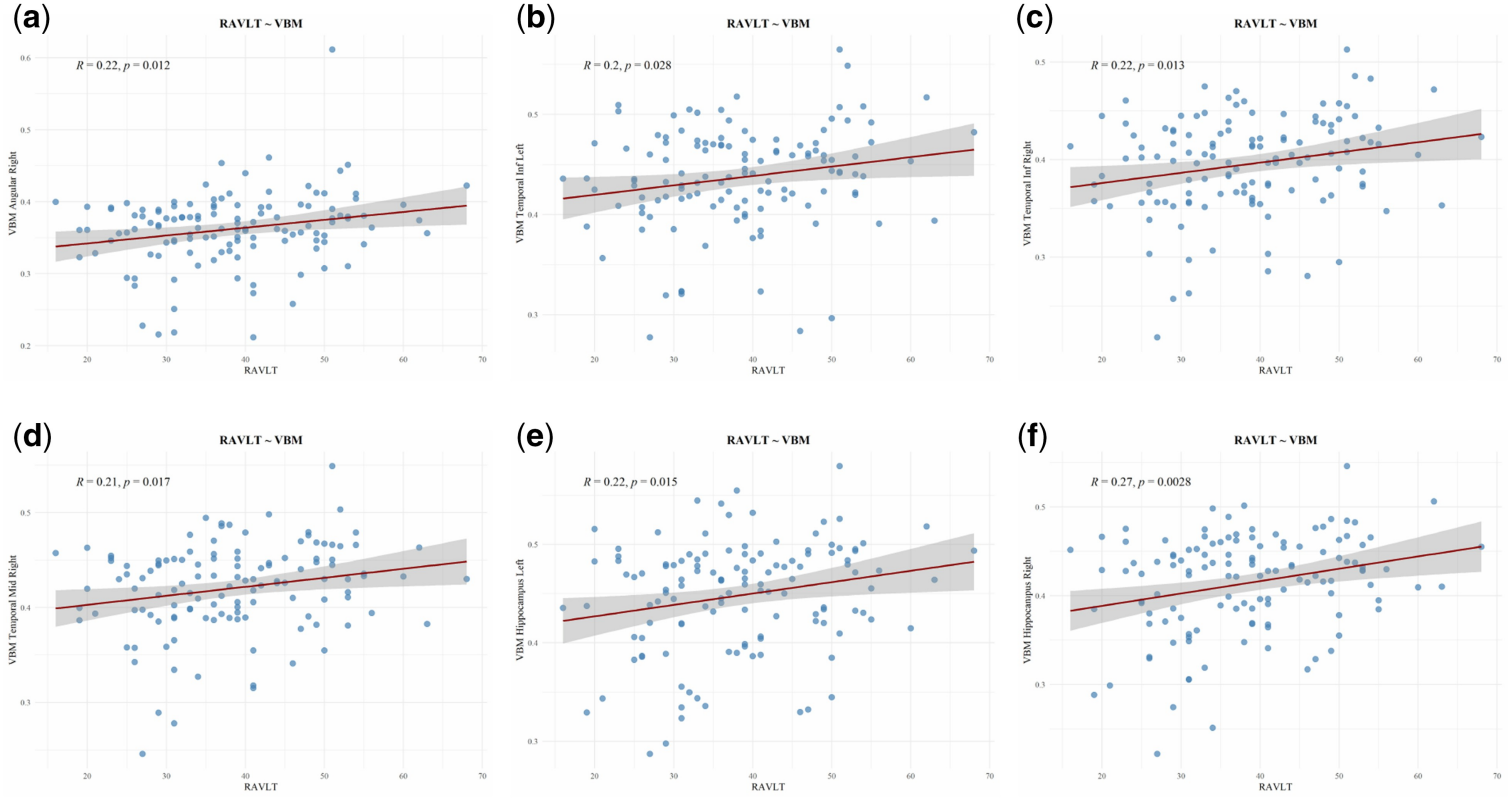
Correlation between the top-selected VBM imaging quantitative traits (QTs) and the clinical cognitive score RAVLT. Subfigures (a–f) respectively illustrate the associations between RAVLT and the regional gray matter volumes of: (a) Right Angular Gyrus, (b) Left Temporal Pole, (c) Right Temporal Pole, (d) Right Temporal Middle Gyrus, (e) Left Hippocampus, and (f) Right Hippocampus.

As depicted in Fig. 7(a)–(f), the leading brain regions selected from voxel-based morphometry (VBM) structural MRI—namely the Angular gyrus, inferior and middle Temporal gyri, and Hippocampus—showed strong associations with both diagnostic categories and cognitive performance. These findings underscored their effectiveness in reflecting disease severity. Importantly, the top neuroimaging features correlated significantly with RAVLT scores, supporting their value as robust biomarkers for cognitive decline.

### 3.5 Mediation analysis for uncovering the pathogenic pathways of Alzheimer’s disease

**Figure 8.**
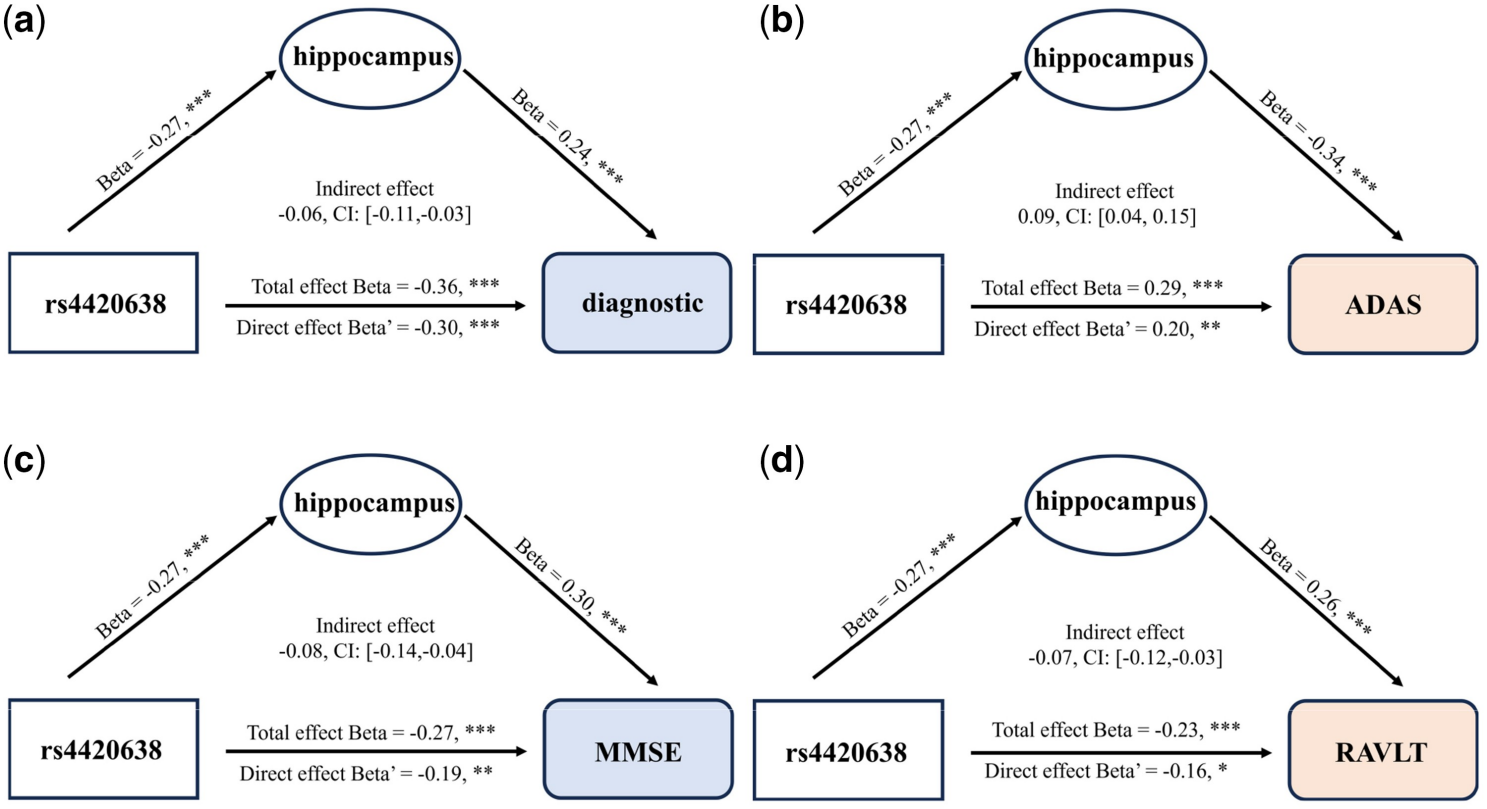
Analysis of the mediating role of endophenotypic traits on diagnostic outcomes for top-selected genetic variants: (a) Diagnostic status, (b) ADAS, (c) MMSE, and (d) RAVLT. *P < .05, **P < .005, ***P < .001.

Focusing on the most prominent genotype–phenotype pair (rs4420638, hippocampal), we conducted mediation analysis to model the causal pathways connecting genetic variation, brain structure, and disease status. In this model, the SNP served as the independent variable, diagnosis as the outcome, and hippocampal volume as the mediator. This approach provided mechanistic insights into how genetic risk factors may influence disease manifestation through structural brain alterations. As shown in Fig. 8, selecting hippocampal volume as the mediator revealed a significant association between rs4420638 and diagnostic outcomes (β = −0.36, *P *< .001), with a portion of this effect mediated by the hippocampus (bootstrapped average causal mediation effect: β = 0.06 [0.11, 0.03]), indicating partial mediation. Similar mediation effects were observed for cognitive measures: the hippocampus partially mediated the effect of rs4420638 on ADAS scores (β = 0.09 [0.04, 0.15]), MMSE scores (β = 0.08 [0.14, 0.04]), and RAVLT scores (β = 0.07 [0.12, 0.03]). These results demonstrated that hippocampal atrophy functioned as a key intermediate phenotype linking genetic risk to both clinical diagnosis and cognitive decline.

### 3.6 Independent experiments

**Figure 9.**
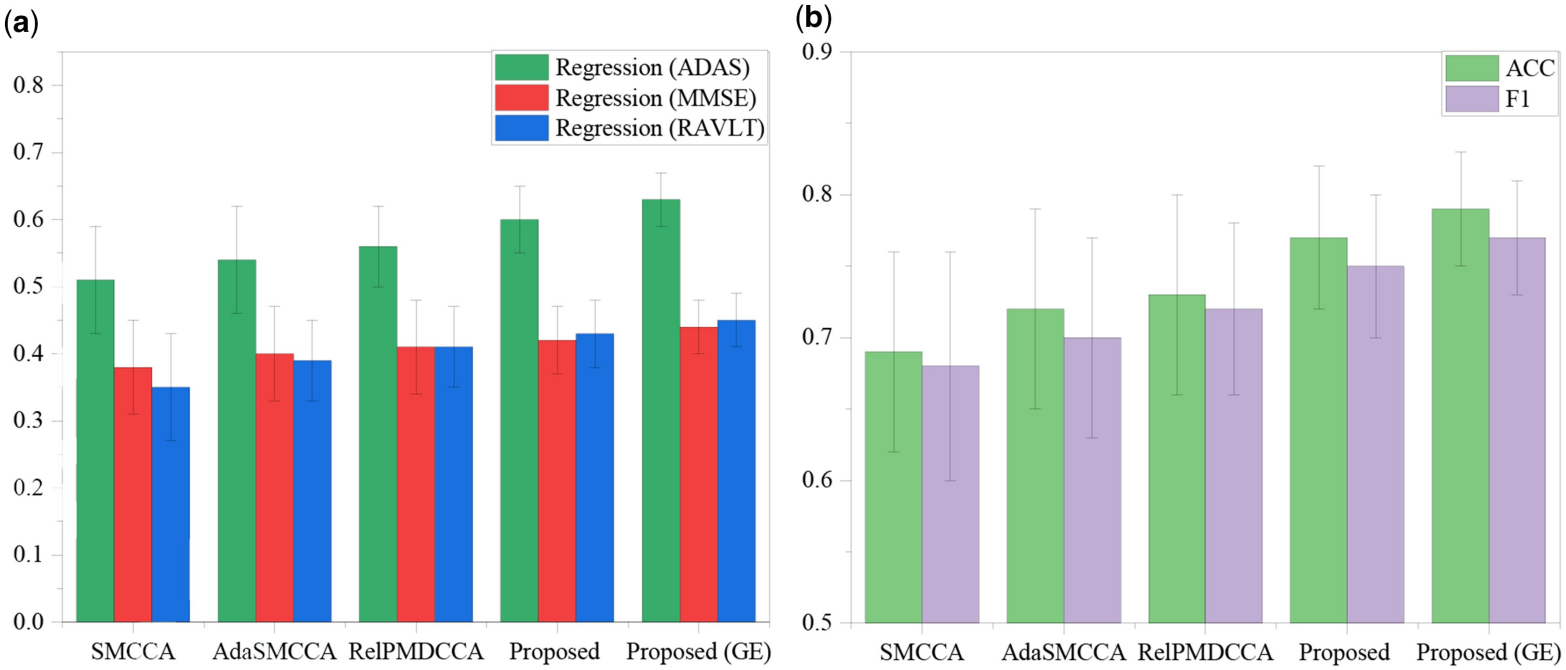
Comparison of testing results for AD diagnosis (ACC, *F*1) and assessment (CCC) on FreeSurfer (a, b) datasets.

To assess the generalizability of our model, we conducted additional experiments using an independent imaging QTs dataset derived from FreeSurfer processing of the ADNI database. In parallel, we also retrieved corresponding genomic, proteomic, and environmental data for the same subjects from the ADNI cohort. The full analytical pipeline was systematically applied to this dataset. Results confirmed that genes such as *APOE*, *TOMM*40, and *APOC*1 contribute to disease phenotypes across multiple brain regions. Moreover, our method identified significant GE interactions, including (rs79429216, motor function), (rs1160984, stroke), and (rs71352241, alcohol abuse). Pa-MACRO also detected key FreeSurfer markers, including hippocampal volume, bilateral entorhinal cortex, mid-temporal regions, and precuneus regions. For plasma biomarkers, nearly all top-ranked features, such as ApoE, MIG, and CRP, were AD-related. Notably, our approach achieved the highest fidelity in clinically relevant brain biomarkers. Collectively, these findings demonstrated the robustness and generalizability of our model across diverse datasets. As illustrated in Fig. 9, Pa-MACRO consistently outperformed baseline approaches on FreeSurfer datasets.

### 3.7 Ablation study

Finally, to investigate the effectiveness of Pa-MACRO, we ran ablation experiments to investigate the impact of the main component on average Prediction (PCCC) and Classification (MACC) tasks.

**Effect of SVR:**  Table 1 reports the test PCCCs and MACCs for all the different choices, revealing that the exclusion of $L_{\text{SVR}}$ resulted in suboptimal performance. This underscored the crucial role of integrating $L_{\text{SVR}}$ in our model.

**Table 1.** Ablation studies on main modules of different design choices from the ADNI-VBM dataset.^a^

*ID*	$L_{\text{SVR}}$	$L_{\text{MABM}}$	$L_{\text{MAJCR}}$	$L_{\text{SCL}}$	PCCC↑	MACC↑
(a)	×	×	×	×	0.41 ± 0.06	0.70 ± 0.08
(b)	✓	×	×	×	0.42 ± 0.07	0.72 ± 0.06
(c)	✓	✓	×	×	0.44 ± 0.07	0.73 ± 0.07
(d)	✓	✓	✓	×	0.46 ± 0.06	0.76 ± 0.06
(e)	✓	✓	✓	✓	**0.48 ± 0.05**	**0.78 ± 0.04**

a “$L_{\text{SVR}}$” denotes sparse variable regularizer. “$L_{\text{SCL}}$” means semi-supervised cooperative learning regularization. Bold indicates the best result. Underline indicates the second-best performance.  ↑ means higher is better.

**Effect of MABM:** The ablation results on PCCCs and MACCs indicated that considering the $L_{\text{MABM}}$ improved disease diagnosis and prediction. This enhancement may stem from the exploration of multimodal pathogenesis, which played a crucial role in enhancing regression and classification.

**Effect of MAJCR:** Pa-MACRO achieved the highest average PCCCs and MACCs with the incorporation of $L_{\text{MAJCR}}$. We attributed this improvement primarily to the unified mutual-assistance framework, which jointly modeled disease detection and severity prediction.

**Effect of SCL:** Finally, as shown in Table 1, the integration of $L_{\text{SCL}}$ into Pa-MACRO led to notable performance gains and a substantial reduction in variance. We attributed these improvements primarily to the effective incorporation of unlabeled data, which enhanced predictive accuracy while promoting model reliability.

## 4 Discussion and conclusions

In this study, we proposed Pa-MACRO, a unified predictive framework integrating multimodal pathogenesis embeddings for neurodegenerative disease diagnosis and prediction. Across two independent neuroimaging datasets, our model consistently achieved significant quantitative improvements over state-of-the-art baselines. On the ADNI-VBM dataset, Pa-MACRO outperformed the strongest baseline by +0.05 (CCC, +11.6%), +0.07 (ACC, +9.9%), and +0.06 (*F*1, +8.6%).

To validate the biological relevance of the identified biomarkers, we conducted comprehensive follow-up analyses. ANOVA confirmed significant differences (*P *< .05) in key genetic and imaging markers, including *APOE* rs429358 (*P *= 7.73 × 10^−10^), *APOC1* rs4420638/rs56131196/rs12721051 (*P *= 1.88 × 10^−10^). Mediation analysis (Fig. 8) revealed that hippocampal volume partially mediated the causal path rs4420638 to diagnosis with an average effect β=−0.06 [−0.11,−0.03], *P *< .001, linking *APOC1* variants to hippocampal atrophy and AD diagnosis.

Gene expression profiling (GTEx + BrainSpan) revealed lifelong high expression of *APOE*, early up-regulation of *TOMM40*, and prenatal/postnatal peaks of *APOC1*, suggesting distinct neurodevelopmental trajectories (Appendix, available as supplementary data at *Bioinformatics* online). PheWAS linked rs4420638 (*APOC1*) to Alzheimer’s disease, parental dementia history, and cardiometabolic traits (cholesterol, hypertension), indicating pleiotropic influence across physiological systems (Fig. 6). Finally, DisGeNET highlighted “Late-Onset Alzheimer’s Disease,” “Dyslipoproteinemias,” and “Autoantibody measurement” as top enriched categories.

Collectively, these findings established both quantitative superiority and pathophysiological interpretability of Pa-MACRO. The framework not only improved predictive accuracy by approximately 10% over leading baselines but also identified mechanistically coherent multimodal biomarkers linking genetic variants (*APOE/APOC1/TOMM40*) to imaging endophenotypes (hippocampal and temporal atrophy) and cognitive decline. By bridging neural circuitry, genetic mechanisms, and predictive modeling, Pa-MACRO provided a reproducible, biologically grounded paradigm for trustworthy AI-driven research, with potential extensions to other neurodegenerative disorders and future integration of additional multimodal datasets for comprehensive disease modeling.

## Supplementary Material

btaf629_Supplementary_Data
