# Supplementary material for: Mutual learning for joint disease detection and severity prediction reveals multimodal pathogenesis for neurodegenerative disorders
Source: Bioinformatics. 2025 Dec 27;42(1):btaf629. doi: 10.1093/bioinformatics/btaf629 (PMC12758602; doi:10.1093/bioinformatics/btaf629)
Supplement: btaf629_Supplementary_Data [file btaf629_supplementary_data.pdf]

Appendix / supplemental material for "**Mutual Learning for Joint Disease Detection and Severity Prediction Reveals Multimodal Pathogenesis for Neurodegenerative Disorders**"

A. Experiments on Real Neuroimaging Genetics Datasets

A.1. ***Application to Alzheimer's Disease***

Dataset: Data used in the preparation of this article were obtained from the Alzheimer's Disease Neuroimaging Initiative (ADNI) database (adni.loni.usc.edu). The ADNI was launched in 2003 as a public-private partnership, led by Principal Investigator Michael W. Weiner, MD. The primary goal of ADNI has been to test whether serial magnetic resonance imaging (MRI), positron emission tomography (PET), other biological markers, and clinical and neuropsychological assessment can be combined to measure the progression of mild cognitive impairment (MCI) and early Alzheimer's disease (AD).

The study utilized a total of 244 participants sourced from the Alzheimer's Disease Neuroimaging Initiative (ADNI) database (adni.loni.usc.edu), consisting of 42 healthy controls (HCs), 137 individuals with mild cognitive impairment (MCI), and 65 subjects clinically diagnosed with Alzheimer's disease (AD). All participants were screened following the ADNI enrollment criteria to ensure diagnostic reliability and imaging consistency.

A comprehensive multimodal dataset was constructed by integrating molecular biomarkers, neuroimaging traits, genetic variants, and environmental factors:

Proteomic markers: Cerebrospinal fluid and plasma proteomic profiles were quantified using the Rules-Based Medicine (RBM) platform. After stringent quality control procedures—removal of low-confidence analytes, outlier detection, and missing rate filtering—146 high-confidence protein markers were retained for downstream analysis.

Neuroimaging phenotypes: Voxel-based morphometry (VBM) was performed on structural MRI scans to extract quantitative imaging traits (QTs) representing regional gray matter density variations across the brain.

In addition, FreeSurfer was employed to derive cortical and subcortical morphometries, including regional cortical thickness, surface area, and volumetric measures, which have been widely validated as sensitive neurodegeneration indicators.

Environmental and clinical risk factors: non-genetic variables were incorporated to account for lifestyle and clinical influences, including age, visual status, body mass index, alcohol consumption, drug sensitivity, blood pressure, smoking history, and history of stroke events. These features help characterize heterogeneous disease risks beyond molecular biomarkers alone.

To further enhance the model's generalizability and biological interpretability, we incorporated unlabeled large-scale SNP and proteomic data obtained from external multi-omics repositories. These unlabeled samples were used during semi-supervised training to improve robustness of disease prediction and stability of biomarker discovery, particularly in high-dimensional settings.

After standard genomic quality control procedures, a total of 10,000 high-quality SNPs were retained. Notably, these included established AD-risk loci such as *APOE*, *APOC1*, and *TOMM40*, ensuring biological relevance for downstream association analysis and interpretation.

To maintain consistency across heterogeneous feature scales, all variables were z-score normalized, which reduces confounding effects from measurement units and improves optimization stability for learning-based models. The final multimodal dataset therefore provides broad coverage of molecular, structural, genetic, and environmental determinants of AD, facilitating comprehensive disease modeling and biomarker identification.

A.2. ADNI Datesets

Data collection and sharing for this project was funded by the Alzheimer's Disease Neuroimaging Initiative (ADNI) (National Institutes of Health Grant U01 AG024904) and DOD ADNI (Department of Defense award number W81XWH-12-2-0012). ADNI is funded by the National Institute on Aging, the National Institute of Biomedical Imaging and Bioengineering, and through generous contributions from the following: AbbVie, Alzheimer's Association; Alzheimer's Drug Discovery Foundation; Araclon Biotech; BioClinica, Inc.; Biogen; Bristol-Myers Squibb Company; CereSpir, Inc.; Cogstate; Eisai Inc.; Elan Pharmaceuticals, Inc.; Eli Lilly and Company; EuroImmun; F. Hoffmann-La Roche Ltd and its affiliated company Genentech, Inc.; Fujirebio; GE Healthcare; IXICO Ltd.; Janssen Alzheimer Immunotherapy Research & Development, LLC.; Johnson & Johnson Pharmaceutical Research & Development LLC.; Lumosity; Lundbeck; Merck & Co., Inc.; Meso Scale Diagnostics, LLC.; NeuroRx Research; Neurotrack Technologies; Novartis Pharmaceuticals Corporation; Pfizer Inc.; Piramal Imaging; Servier; Takeda Pharmaceutical Company; and Transition Therapeutics. The Canadian Institutes of Health Research is providing funds to support ADNI clinical sites in Canada. Private sector contributions are facilitated by the Foundation for the National Institutes of Health (www.fnih.org). The grantee organization is the Northern California Institute for Research and Education, and the study is coordinated by the Alzheimer's Therapeutic Research Institute at the University of Southern California. ADNI data are disseminated by the Laboratory for Neuro Imaging at the University of Southern California.

B. Follow-up analyses for biomarker discovery

B.1. ***Follow-up analyses: Gene-set analyses***

To evaluate the clinical relevance of the identified SNPs, we conducted a one-way analysis of covariance (ANOVA) to assess their association with diagnostic phenotypes. As expected, all selected SNPs showed statistically significant effects (p<0.05), underscoring their potential roles in disease pathology. Notably, the most significant loci included rs429358 in *APOE* ($p = 7.73 \times 10^{-10}$), and multiple variants within *APOC1* (e.g., rs4420638, rs56131196, rs12721051, all

$p = 1.88 \times 10^{-9}$), these findings validated the robustness of Pa-MACRO in isolating biologically relevant markers from high-dimensional genomic data.

To further investigate gene-level contributions of the identified loci, we performed gene set enrichment analysis (GSEA) using the MAGMA analytical framework. This gene-centric method applied a multiple linear regression model based on principal components, which were extracted from the linkage disequilibrium (LD) patterns of SNPs located within each gene. Following this, we employed Fisher's combined probability test to estimate the overall significance of gene–phenotype associations.

The results revealed highly significant associations for several key genes: $APOE$, $APOC1$. These genes were well-documented in Alzheimer's disease research, and their prominence in our findings confirms the model's capability to uncover meaningful genetic contributors amid a vast omics landscape. Collectively, these results demonstrated that by integrating a mutual-assistance framework, Pa-MACRO significantly advanced beyond conventional approaches in identifying reliable and mechanistically relevant biomarkers for neurodegenerative disorders.

B.2. ***Follow-up analyses: Functional mapping***

To validate the causal relevance of identified biomarkers and enhance biological interpretability, we utilized the Functional Mapping and Annotation (FUMA) platform to perform functional mapping, variant prioritization, and annotation of GWAS summary statistics. Based on the 1000 Genomes Phase 3 reference panel, independently significant SNPs were determined using a genome-wide significance threshold ($p < 5 \times 10^{-8}$) and filtered for independence within a 1-Mb window. From this set, lead SNPs were subsequently extracted, confirming their association with Alzheimer's disease. After the analysis, multiple loci reached genome-wide significance in relation to diagnostic categories, with the most prominent signals observed at $APOE$, $APOC1$, and $TOMM40$. These results closely matched those identified by Pa-MACRO, offering strong external validation for our approach. In particular, rs429358—positioned within $APOE$ stood out as the lead SNP, demonstrating the strongest association with AD. This emphasized the robustness and interpretability of our method in uncovering genetic variants that are both biologically meaningful and clinically relevant.

B.3. ***Follow-up analyses: Gene expression analyses***

To better interpret the transcriptomic relevance of the identified loci, we conducted gene expression profiling through the GENE2FUNC module. This allowed us to examine expression patterns of genes mapped from the top-ranking SNPs across diverse human tissues. The analysis incorporated RNA-seq datasets from GTEx (v8), covering 54 tissue types, as well as BrainSpan, which included 29 developmental stages of the human brain. We visualized the results using heatmaps that represented average normalized expression levels, offering an integrated overview of spatial and temporal gene activity.

As shown in Figure 1, the expression profiles of genes linked to the top SNPs, such as $APOE$, $APOC1$, and $TOMM40$ were visualized across both adult and

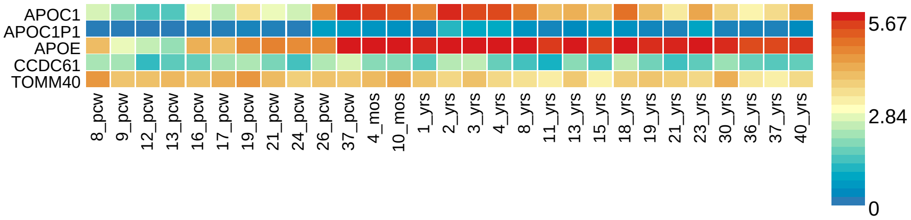

(a)

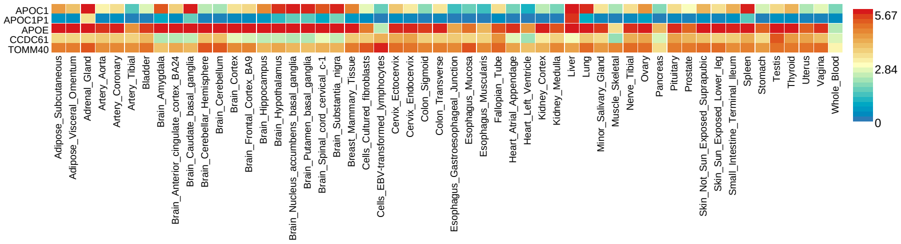

(b)

Figure 1: Heatmaps depicting normalized gene expression levels, derived from zero-mean normalization of log2-transformed values, are shown for the prioritized genes. The lower section displays data from GTEx v8 RNAseq, whereas the upper section presents BrainSpan data.

developing brain tissues. The upper panel illustrates expression levels from GTEx, revealing distinct regional and tissue-specific transcriptional signatures. $APOE$ demonstrated consistently high expression across all life stages, indicating its ubiquitous role in brain function. In contrast, $APOC1$ showed elevated expression during the late prenatal and early postnatal periods. $TOMM40$ exhibited heightened expression during early developmental stages, which may be implicated in neurodevelopmental trajectories contributing to neurodegenerative risk.

These expression dynamics underscored the biological validity of our prioritized loci and further reinforced the capability of Pa-MACRO to identify genetically and functionally relevant biomarkers across the human lifespan.

B.4. ***Follow-up analyses: Association enrichment analysis***

We conducted enrichment analysis on the top-ranked SNPs using DisGeNET ontology categories through the Metascape platform. DisGeNET aggregated gene–disease associations from curated databases, GWAS repositories, animal studies, and peer-reviewed publications, offering an integrative view of the genetic landscape of human disorders. As presented in Figure 2, this analysis revealed significant enrichment in several disease-related biological pathways, such as "Autoantibody Measurement," "Late-Onset Alzheimer's Disease," "Dyslipoproteinemias," "Focal Onset Alzheimer's Disease," and "Acute Confusional Senile Dementia." These results shed light on the molecular basis of

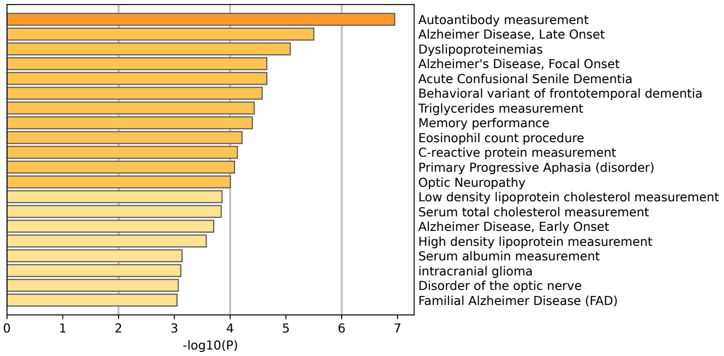

Figure 2: Summary of enrichment analysis in DisGeNET.

Alzheimer's pathology and underscored the therapeutic potential of the identified genetic biomarkers associated with AD.

B.5. ***Correlation Between Imaging-Based Biomarkers and Diagnosis Assessments***

To facilitate clinical application of our findings, we examined the relationships between top neuroimaging-derived phenotypes and cognitive function in individuals at risk for AD. Specifically, we analyzed correlations between the identified imaging biomarkers and diagnosis assessment. As depicted in Figures 3, the leading brain regions selected from voxel-based morphometry (VBM) structural MRI—namely the Angular gyrus, inferior and middle Temporal gyri, showed strong associations with both diagnostic categories. These methodologies enhance model performance while advancing our understanding of the neural circuits underlying diagnosis.

B.6. ***The robustness of the Pa-MACRO model***

To further evaluate the robustness of the proposed model, we assessed its prognostic performance under varying levels of feature perturbation. Gaussian noise $\mathcal{N}(\mathbf{0}, \sigma\mathbf{I}_{n\times n})$ was introduced into the multi-omics datasets, with noise levels set at $\sigma = 0.01$ (noisy1), 0.05 (noisy2), and 0.1 (noisy3), corresponding to increasing perturbation intensities. The model was subsequently trained and evaluated on these perturbed datasets. As shown in Figures 4, the model consistently exhibited stable and competitive performance across all noise levels, demonstrating its robustness against input variability. This resilience was largely attributed to the joint optimization strategy employed for biomarker selection and disease prediction, which mitigated the risk of overfitting in high-dimensional multimodal biomedical data.

To evaluate the robustness and reproducibility of our biomarker identification framework, we performed a consistency analysis across multiple experimental runs. Specifically, we compared the top 50 biomarkers selected in each run,

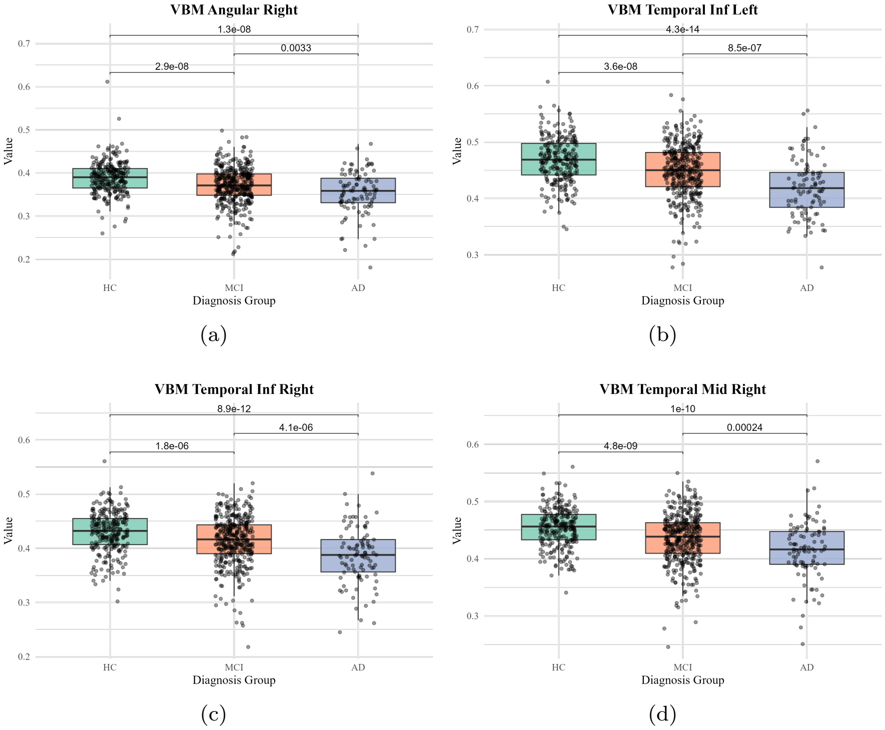

Figure 3: Each boxplot displays the distribution of the top-ranked imaging QTs (VBM-sMRI) for the respective diagnostic categories. The y-axis represents the selected imaging QTs, while the x-axis denotes the diagnostic groups: HC, MCI, and AD. Statistical significance (p-values) for comparisons between pairs of diagnostic groups are also provided.

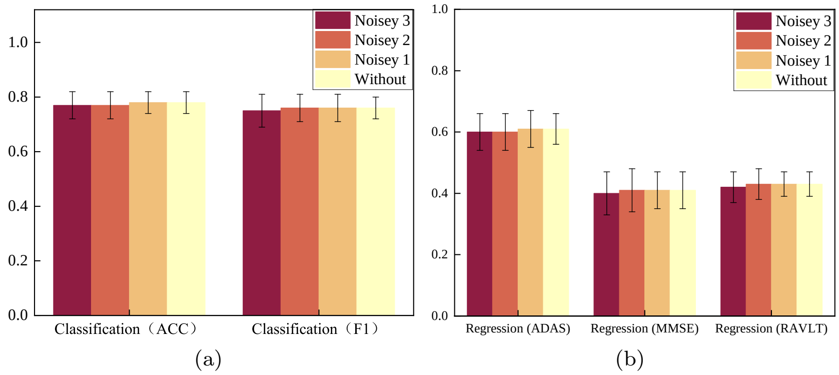

Figure 4: Performance comparison under varying noisy rates on disease detection (a) and severity prediction (b) in multi-omics datasets.

where each iteration used a different training–testing split. Consistency was measured by the average pairwise overlap of biomarkers across these runs.

We conducted 50 independent experiments using varied data partitions from the ADNI VBM and FreeSurfer datasets. The mean overlap rates reached 98% for VBM and 96% for FreeSurfer, respectively. These findings demonstrated the high stability and reproducibility of our biomarker discovery method, even under varying experimental conditions and across different datasets.

Symbol / Parameter	Definition / Meaning	Dimension / Description
$\mathbf{X} \in \mathbb{R}^{n \times p_1}$	SNP genotype matrix	n: number of subjects, p_1: number of SNPs
$\mathbf{Y} \in \mathbb{R}^{n \times p_2}$	Imaging phenotype matrix	p_2: number of imaging features
$\mathbf{C} \in \mathbb{R}^{n \times p_3}$	Proteomic feature matrix	p_3: number of proteomic features
$\mathbf{E} \in \mathbb{R}^{n \times p_4}$	Environmental exposure matrix	p_4: number of environmental variables
$\mathbf{z} \in \mathbb{R}^{n}$	Continuous clinical outcome	—
$\mathbf{d}$	Diagnostic status (categorical variable)	—
$\mathbf{U} = [\mathbf{u}_1, \mathbf{u}_2, \mathbf{u}_3]$	Latent projection coefficients for SNPs	$p_1 \times r$
$\mathbf{V} = [\mathbf{v}_1, \mathbf{v}_2, \mathbf{v}_3]$	Latent projection coefficients for proteomic features	$p_3 \times r$
$\mathbf{W} = [\mathbf{w}_1, \mathbf{w}_2, \mathbf{w}_3]$	Latent projection coefficients for imaging features	$p_2 \times r$
$\mathbf{Q} \in \mathbb{R}^{p_1 \times p_4}$	Interaction coefficient matrix between SNPs and environment	—
$\mathbf{X}', \mathbf{C}'$	Unlabeled SNP and proteomic data	$n_{\text{unlabeled}} \times p_1$, $n_{\text{unlabeled}} \times p_3$
d_{ik}	Predicted disease status for subject i and class k	—

Table 1: Explanation of all parameters used in the three MA parts.

B.7. ***Clarification of Parameters in the MA Framework.***

To improve clarity and readability, we had provided a comprehensive summary of all symbols and parameters used in the three Mutual-Assistance (MA) components (see Table 1). In particular, the latent projection vector $\mathbf{u}$ is decomposed into three task-specific components $\mathbf{u}_1$, $\mathbf{u}_2$, and $\mathbf{u}_3$, corresponding respectively to: (i) the association between SNPs and imaging phenotypes, (ii) the association between SNPs and proteomic biomarkers, and (iii) the joint contribution of multimodal features to clinical outcomes. This decomposition enabled the model to capture both shared and task-specific effects across modalities, thereby enhancing interpretability and avoiding overly restrictive shared-parameter assumptions.
